# LIFE 4 Pollinators’ platform: How citizen science can help monitoring plants and pollinators

**DOI:** 10.1093/aobpla/plaf023

**Published:** 2025-04-18

**Authors:** Fortunato Fulvio Bitonto, Roberto Costantino, Marta Barberis, Gherardo Bogo, Daniele Birtele, Giacomo Cangelmi, Matteo Dal Cin, Jelle Devalez, Lucia Lenzi, Serena Magagnoli, Alessio Minici, José María Sánchez, Emanuele Luigi Zenga, Laura Bortolotti, Luis Navarro, Theodora Petanidou, Fabio Sgolastra, Anna Traveset, Marta Galloni

**Affiliations:** Department of Biological, Geological and Environmental Sciences, University of Bologna, Via Irnerio 42, 40126 Bologna, Italy; Department of Agriculture and Food Sciences, University of Bologna, Viale Giuseppe Fanin 42, 40127 Bologna, Italy; Regional Museum of Natural Sciences of Torino, Via Giovanni Giolitti 36, 10123 Turin, Italy; Department of Biological, Geological and Environmental Sciences, University of Bologna, Via Irnerio 42, 40126 Bologna, Italy; CREA Research Centre for Agriculture and Environment, Via di Corticella 133, 40128 Bologna, Italy; Reparto Carabinieri Biodiversità di Roma, Via Canale della Lingua 74, 00124, Roma, Italy; Department of Life, Health & Environmental Science, University of L’Aquila, Via Vetoio 40, 67100 L’Aquila, Italy; Department of Life Sciences, University of Siena, Via Pier Andrea Mattioli 4, 53100 Siena, Italy; Laboratory of Biogeography and Ecology, Department of Geography, University of the Aegean, University Hill, 81100 Mytilene, Greece; Department of Agriculture and Food Sciences, University of Bologna, Viale Giuseppe Fanin 42, 40127 Bologna, Italy; Department of Agriculture and Food Sciences, University of Bologna, Viale Giuseppe Fanin 42, 40127 Bologna, Italy; Department of Computer Science, University of Verona, Strada le Grazie 15, 37134 Verona, Italy; Department of Plant Biology and Soil Science, Faculty of Sciences, University of Vigo, Campus Lagoas-Marcosende, 36310 Vigo, Spain; CREA Research Centre for Agriculture and Environment, Via di Corticella 133, 40128 Bologna, Italy; Department of Agricultural, Forest, and Food Sciences, University of Turin, Largo Paolo Braccini 2, 10095 Grugliasco, Torino, Italy; CREA Research Centre for Agriculture and Environment, Via di Corticella 133, 40128 Bologna, Italy; Department of Plant Biology and Soil Science, Faculty of Sciences, University of Vigo, Campus Lagoas-Marcosende, 36310 Vigo, Spain; Laboratory of Biogeography and Ecology, Department of Geography, University of the Aegean, University Hill, 81100 Mytilene, Greece; Department of Agriculture and Food Sciences, University of Bologna, Viale Giuseppe Fanin 42, 40127 Bologna, Italy; Global Change Research Group, Mediterranean Institute for Advanced Studies, C/Miguel Marquès 21, 07190 Esporles, Mallorca, Balearic Islands, Spain; Department of Biological, Geological and Environmental Sciences, University of Bologna, Via Irnerio 42, 40126 Bologna, Italy

**Keywords:** participatory web platform, threatened species, alien species, conservation, plant-pollinator interactions

## Abstract

Plant diversity is critical to ensure the future of humanity, as it provides essential ecosystem services and functioning. As recent estimates showed that animal-mediated pollination is crucial for the reproduction of approximately 90% of flowering plants, playing an essential role in maintaining biodiversity and agricultural productivity, effort to preserve plants cannot be disjoined from pollinator conservation initiatives. Despite their importance, pollinators have experienced alarming declines. The LIFE 4 Pollinators project was launched to involve people protecting wild bees and other pollinators in the Mediterranean. This study presents data collected through the project’s web-platform, where users uploaded over 2,000 photographs of plant-pollinator interactions between 2021 and 2024. The dataset focuses on the identification of flower-visiting insects and plants, and the current study gives emphasis to citizen scientists’ ability to identify plants and pollinators. 1,407 photo-records were analysed, revealing that bees and beetles were the most frequent pollinators, with plants of the Asteraceae and Cistaceae families being the most recorded. Users correctly identified 93.7% of insect taxonomic aggregations and 74.2% of plant species. The study also highlights the recording of threatened, alien, and invasive species, including the vulnerable *Callicera spinolae* and the invasive *Vespa velutina*. The plant-pollinator network analysis supports the floral syndrome concept, with floral morphologies like ‘Head’ and ‘Disk’ attracting a wide range of pollinators. The results indicate that citizen science contributes to the identification and monitoring of pollinators, generating knowledge that may be key to the conservation of these organisms and to better understand plant-pollinator interactions. Data collection through citizen-generated photographs allows to significantly expand the geographic area and the magnitude of studies, facilitating large-scale analyses that would be difficult to achieve with traditional monitoring methods. These findings provide a useful basis for future conservation initiatives and the development of policies aimed at mitigating pollinator decline.

## Introduction

The last century has seen an unprecedented loss of habitat and biodiversity, primarily driven by changes in land use (Haddad *et al*. 2015). Coupled with climate change, this has led to the erosion of genetic diversity (e.g. Laikre *et al*. 2020; Des Roches *et al*. 2021), decline in species richness (Tilman *et al*. 2017), and reduction of ecosystem services (Cardinale *et al*. 2012). The loss of plant diversity, in particular, has critical consequences for the future of humanity (Raven and Wagner 2021). Beyond their essential role in nutrient sequestration and habitat shaping (Giam *et al*. 2010), in fact, plant diversity supports human food security (Kier *et al*. 2005), provides key ecological services (e.g. Mace *et al*. 2012; Molina-Venegas *et al*. 2021), and not least sustains other living organisms (Huston 1994; Primack and Corlett 2005).

Despite their importance, recent assessment on the extinction risk of European species has shown that between 7% and 9% of the European vascular plant species are threatened, and up to 84% of these species have not yet been assessed in the global Red List (Holz *et al*. 2022). Such an assessment is crucial and urgent, as not all plant species respond equally to the rapid habitat loss and climate change. Their vulnerability, in fact, is influenced either by factors intrinsic to their biology (e.g. Hamrick and Godt 1996; De Kort *et al*. 2021) or exacerbated by fragmentation- and climate-driven loss of mutualistic partners, such as pollinators (e.g. Bennett *et al*. 2020; Rodger *et al*. 2021). As recent estimates show that up to 90% of flowering plant species depend on animal pollination (Tong *et al*. 2023), efforts aimed to preserve plant diversity cannot be disjoined by initiatives targeting pollinator conservation, and *vice versa*. For their part, pollinators are widely recognized for providing an essential ecosystem benefit across both natural and agricultural landscapes (Potts *et al*. 2016). The process of pollination is crucial for sustaining biodiversity among wildflower species and indirectly supports the survival of other groups reliant on floral resources, including herbivorous and fruit- and seed-eating animals (Potts *et al*. 2006). Pollinators fulfil an indispensable function in most terrestrial ecosystems, supporting wild plant communities (Ashman *et al*. 2004; Vamosi *et al*. 2006) as well as enhancing agricultural yields (Klein *et al*. 2007; Ricketts *et al*. 2008). Globally, over 75% of key crops depend on animal pollination (Klein *et al*. 2007; Aizen *et al*. 2019). Their services are estimated to be equivalent to billions of dollars in global crop productivity, significantly contributing to nutritional security worldwide (Chaplin-Kramer *et al*. 2014). In Europe, pollinators are primarily insects, including managed and wild bees, wasps, butterflies, moths, beetles, hoverflies, bee flies, and other fly species (Potts *et al*. 2016).

Like plants, despite their importance, pollinators have been undergoing an alarming and severe decline over the past few decades (Wagner 2020). Although data are available only for some pollinator groups and a few global regions, there is clear evidence that several species have reduced their geographical ranges, some have gone extinct, and many others show declines in local abundance (Potts *et al*. 2010, 2016). However, investigations on pollinator abundance show geographical and temporal biases. In fact, studies have predominantly focused on human-modified ecosystems in mid-latitude regions, particularly in Western Europe and North America, where pollinator losses are more pronounced (Ghazoul 2005; Winfree *et al*. 2009; Potts *et al*. 2010). Conversely, evidence from less anthropized environments, such as protected natural areas, showed occasional site-specific positive trends in their abundance, suggesting greater complexity in this phenomenon at different geographical scales (e.g. Herrera 2019).

European red lists of the three main groups of pollinating insects indicate that 9% of bee species (Nieto *et al*. 2014), 9% of butterfly species (Van Swaay *et al*. 2010), and 37% of hoverfly species (Vujić *et al*. 2022) are threatened. In addition, the biology, distribution, and ecology of many species are poorly known, such in the case of bees, for which more than 50% of taxa are considered as ‘Data Deficient’ (Nieto *et al*. 2014), making data collection necessary to address this gap, especially in Natura 2000 sites (Popescu *et al*. 2014; European Parliament 2023).

In this context, particular emphasis has been given to citizen engagement. Citizen Science (hereafter abbreviated as ‘CS’) involves the active participation of the general public in research activities across various disciplines (Vohland *et al.* 2021). CS projects engage citizens as contributors, collaborators, or project leaders, allowing them to play a crucial role in generating new knowledge or understanding (ECSA 2015). Through the collection of ecological data, they can answer research questions, enhance learning and awareness, foster stewardship, and even influence conservation actions and policies (e.g. Dibner and Pandya 2018; Irwin 2018; Aavik *et al*. 2025). Several CS projects focusing on pollinators have been implemented in recent years at local, national, or international levels. Examples include the Bumblebee Conservation Trust’s ‘Bee Walk’ in the UK (Bates *et al*. 2015), ‘Spipoll’ project in France (Griffiths-Lee *et al*. 2022), and ‘Beewatching’ in Italy (Flaminio *et al*. 2021; Bortolotti and Galloni 2025).

The LIFE 4 Pollinators project (LIFE18/GIE/IT/000755) ‘Involving people to protect wild bees and other pollinators in the Mediterranean’ aims to enhance the conservation of pollinating insects and entomophilous plants across the Mediterranean region by fostering a virtuous cycle that promotes progressive changes in anthropogenic practices currently threatening wild pollinators. Beyond the multiple materials produced (including simplified field guides, animation videos, digital tools, and specific handbooks), citizen science (bioblitzes) and didactic activities, trainings to key stakeholders and dissemination events have been carried on, to raise awareness in four European countries: Italy, Greece, Spain, and Slovenia (https://www.life4pollinators.eu/).

The aim of this study is to validate the potential of a CS approach in deepening our knowledge in plant-pollinator interactions. To do so, we analyzed the records uploaded to the LIFE 4 Pollinators web platform (https://www.life4pollinators.eu/en/submission), evaluating the capacity of citizens to identify entomophilous plants and flower insect visitors and improving available information on flower-insect interactions. Mutualistic interactions between pollinators and plants form complex networks, whose analysis can help us understand ecological and functional roles within the communities, offering hints that might be useful for conservation purposes (Borchardt *et al*. 2021; Fisogni *et al*. 2021; Lanuza *et al*. 2025). Our study demonstrates that even data collected by citizen scientists can provide valuable insights into plant-pollinator interactions across broad and local scales, increasing knowledge on the ecological function of community components, including species of conservation concern (with Ons island, Spain, presented as case study).

## Materials and methods

### Platform and data

As part of the LIFE 4 Pollinators project, a web platform was developed in 2021 to collect photographs of insects visiting flowers (https://www.life4pollinators.eu/en/submission).

The platform was advertised in different ways and occasions, and in particular during 27 bioblitz events organized throughout project implementation (from 2021 to 2024), 23 being held within 18 different Natura 2000 sites, with an overall participation of about 800 people (Supplementary Table S7). The platform is freely accessible to the public and, to encourage engagement, no registration and no personal data (age, educational level, employment status…) are required to send photo-records. Users can upload photographs depicting insects visiting flowers (hereafter referred to as ‘observations’) and possibly provide additional details.

The primary details to be recorded include the date of the observation, geographical coordinates, landscape, pollinator behaviour, plant abundance, and site protection status. Users can also try to identify the observed organisms, by selecting the pollinator taxonomic aggregation from a given list of alternative choices (bee, wasp, beetle, butterfly, moth, hoverfly, and bee fly) and/or by writing the name of the pollinator and that of the plant (at the taxonomic level that they choose). Additionally, users may voluntarily provide a nickname and email address.

In the current study, focused mainly on citizens’ ability to identify plants and insects, the parameters landscape, pollinator behavior, and plant abundances have not been considered.

### Taxonomic identification

**Figure 1.**
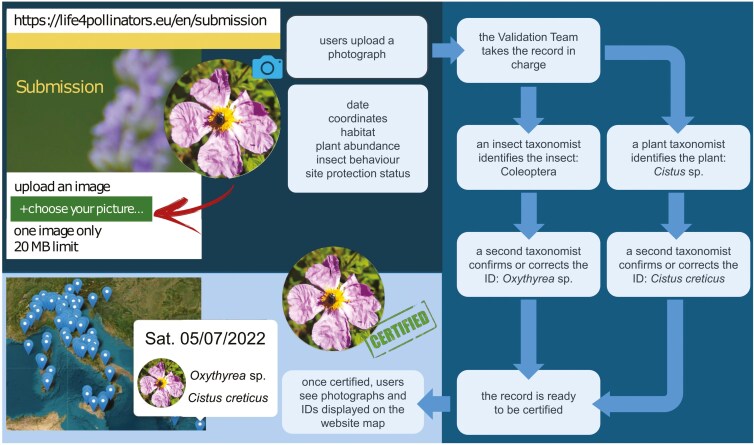
Flowchart illustrating the validation process following photograph upload on the LIFE 4 pollinators web platform.

A team of expert taxonomists identified the observations (hereafter referred to as ‘photo records’ or simply ‘records’) uploaded to the platform to the lowest possible taxonomic level. The identification process consisted of two steps: first, a team member determined the insect and/or plant; then, a second expert confirmed the accuracy or adjusted the identification if necessary. Once verified, the images along with their taxonomic information were displayed publicly on the website map (https://www.life4pollinators.eu/en/map). The validation process is illustrated in Figure 1.

Plant nomenclature was checked using the Plants of the World Online database (https://powo.science.kew.org). Plant taxa were categorized by floral shapes (Bell, Brush, Disk, Disk-Tube, Flag, Funnel, Gullet, Head, Lip, Tube) according to Faegri and Van Der Pijl (1979).

For Hymenoptera Apoidea Anthophila, (bees) we followed Ghisbain *et al*. (2023); Diptera species names (bee flies and hoverflies) were verified through Systema Dipterorum (http://www.diptera.org); Lepidoptera Papilionidae (butterflies) names followed Wiemers *et al*. (2018). Names for Coleoptera, Hymenoptera Vespidae (wasps), and non-Papilionidae Lepidoptera (moths) were checked using the Fauna Europea dataset (de Jong 2016) and the EASIN database for alien species in Europe (https://easin.jrc.ec.europa.eu/easin).

### Elaboration of dataset and species status

On May 31st, 2021 the dataset comprising all records uploaded since the first entry on May 15th, 2021 was downloaded, covering a total of 37 months. The most recent photo is dated May 30th, 2024. Analyses were exclusively focused on plant-pollinator interactions, so all photo records not meeting this criterion, such as pictures of insects on leaves or those of plants alone, were excluded.

Photographic records were used to obtain descriptive networks of plant-pollinator interactions: a qualitative network was constructed to visualize the relationships between plant floral shapes (*sensu*  Faegri and Van Der Pijl 1979) and broad insect taxonomic aggregations (see above), while a plant-pollinator network of Ons Island (ES0000254) was constructed based on species-level identified photo records gathered during a bioblitz conducted in May 2023, where the platform was used to upload the collected data.

The conservation status of plant and pollinator species was checked at European level, through the IUCN Red Lists (www.iucnredlist.org). Alien species status was checked through the European Alien Species Information Network (https://easin.jrc.ec.europa.eu/easin), while invasiveness was verified by consulting the Global Invasive Species Database (https://www.iucngisd.org/gisd) and the IPBES Invasive Species Report (Roy *et al*. 2023).

### Data analysis

Statistical analyses were carried out using R software, version 4.4.1 (R Core Team 2024). Specifically, bar charts and maps were created using various functions from the ‘ggplot2’ package (Wickham 2016). The percentage of photo records within Natura 2000 sites was calculated using QGIS software, version 3.36 (QGIS 2024), by applying the intersection function between the observation coordinates and the Natura 2000 network (European Environment Agency 2022). Boschloo’s test was chosen to compare the correctness of users’ identifications between the functional groups (Boschloo 1970). The significance level was set at 0.05, and the *exact.test* formula of ‘Exact’ package was used (Calhoun and Calhoun 2016).

To account for multiple comparisons and control the false discovery rate, we applied the Benjamini Hochberg, and Yekutieli (BHY) method (Benjamini and Hochberg 1995; Benjamini and Yekutieli 2001) to adjust all p-values. This correction was implemented using the p-adjust function from the ‘Stats’ package in R (R Core Team 2024).

Lastly, plant–pollinator networks were constructed using the *plotweb* function from the ‘Bipartite’ package (Dormann *et al*. 2008).

## Results

### Photo records

**Figure 2.**
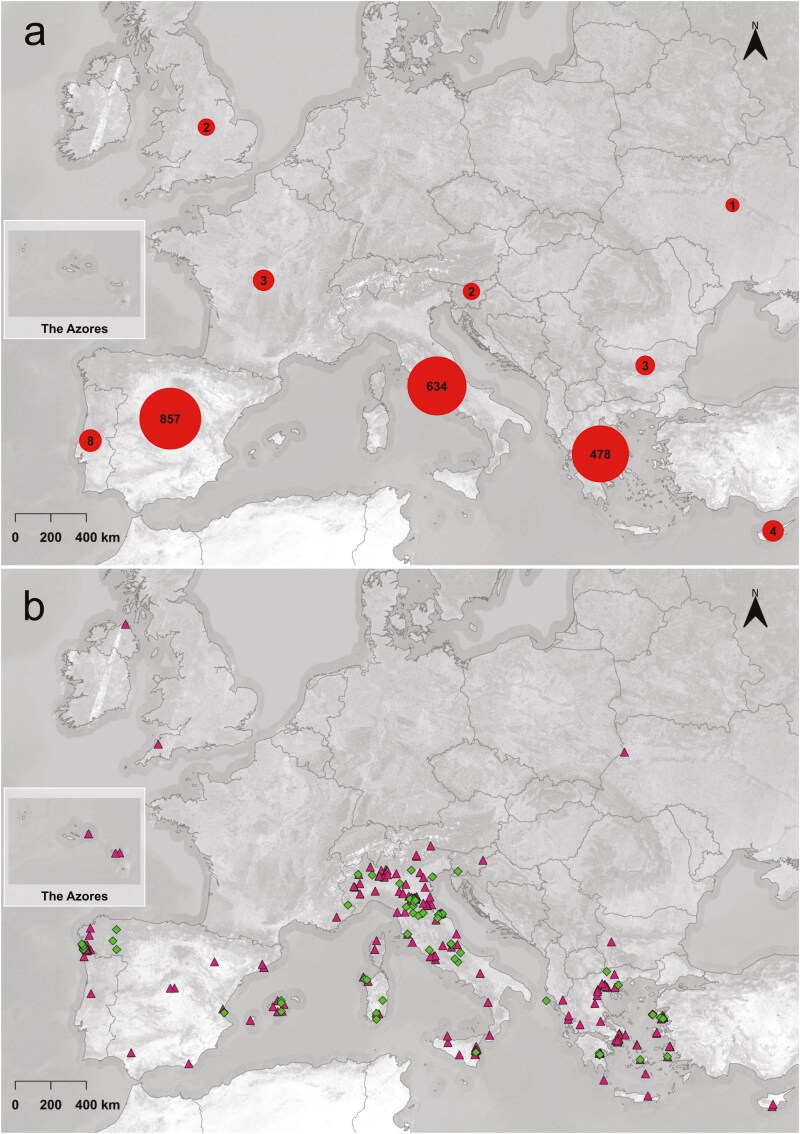
Map of observation counts for each European country (*n *= 1992). The total number of observations for each country is displayed in figure a), location within (diamonds) or outside (triangles) Natura 2000 sites is shown in figure b).

A total of 2094 photo records were uploaded to the platform throughout 3 years of project implementation: 1992 (95.1%) from 10 European countries (Fig. 2), while the remaining 99 (4.7%) were without coordinates. Of the total, 978 (49.1%) are located within 71 Natura 2000 areas in project’s partners’ countries: Greece, Italy, Slovenia, and Spain (Supplementary Table S1). The three countries with the most photo records were Spain (857 out of 1,992; 43.0%), Italy (634; 31.8%), and Greece (478; 24.0%).

Many records were uploaded thanks to citizen engagement during bioblitzes. However, these events often took place in remote locations not served by internet connection. Moreover, the only indication given to volunteer citizens was to photograph and upload insect-flower interactions (possibly adding additional environmental information, following online instructions), so the data gathered should be considered the result of opportunistic collection.

**Figure 3.**
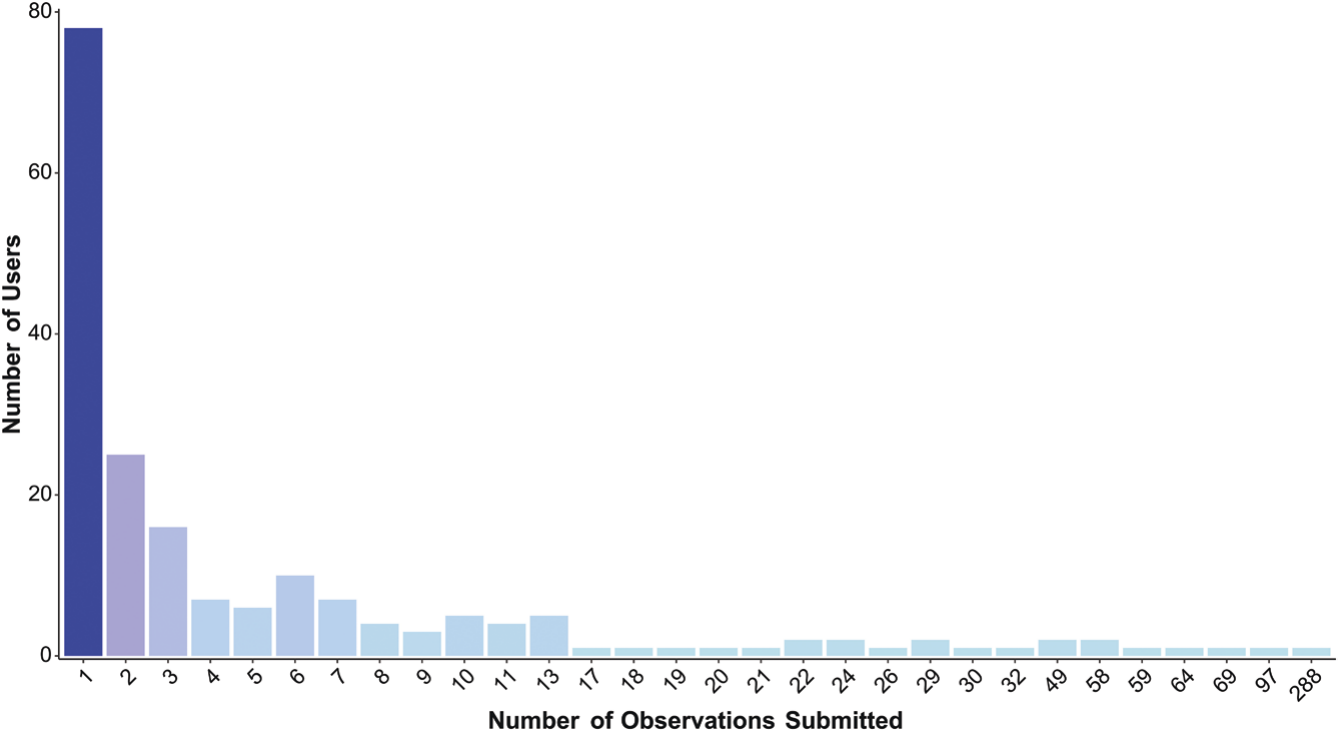
Number of observations uploaded by individual non-anonymous users.

A total of 193 users (Fig. 3) entered contact details, uploading 1695 photo records (80.9%), while the remaining 399 photo records (19.1%) were uploaded anonymously. Among the non-anonymous contributors, most people (161 out of 193; 83.4%) uploaded between 1 and 10 pictures. The highest number of photo records by a single user is 288 (an observer from Sicily, Italy).

**Figure 4.**
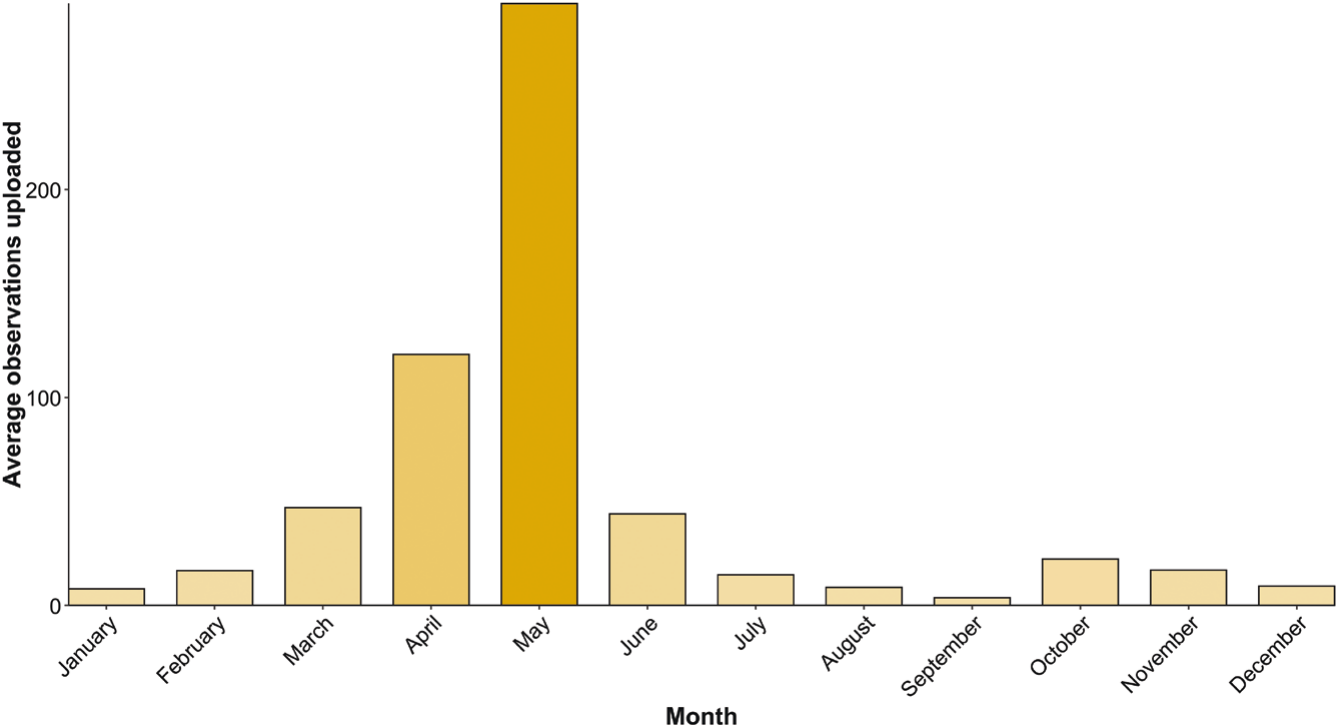
Mean monthly photo records uploads (2021–2024).

More than 80% of the photo records uploaded between 1st May 2021 and 31st May 2024, were uploaded during the spring and early summer months (March to June), with a peak in May (290 on average) (Fig. 4).

**Figure 5.**
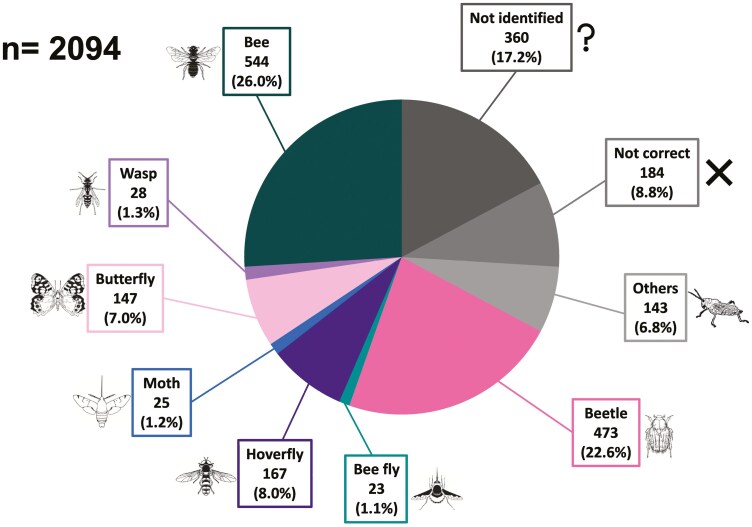
Pie chart displaying the percentages and abundances of the photo-records categorized as: (i) taxonomic aggregations foreseen by the LIFE 4 Pollinators project (Bee, Wasp, Butterfly, Moth, Hoverfly, Bee fly, Beetle), (ii) non-focal insect groups (Others), (iii) lacking insect-flower interaction (Not correct), (iv) yet to be identified at the date of 31 May 2024 (not identified). Insect illustrations obtained from http://divulgare.net.

We analysed 1407 out of the original 2094 records, excluding those pictures lacking insect-flower interaction (184, 8.8%), those yet to be identified at the date of May 31st, 2024, (360, 17.2%), and those depicting insects different from our target insect taxonomic aggregations (143, 6.8%) (Fig. 5, Supplementary Table S2).

### Pollinators

Of the 1407 insect records, 844 (60%) photographs allowed identification at the species level, while 455 (32%) and 85 (6%) were identified at the genus and family levels, respectively. The remaining 23 (2%) were classified into broad taxonomic aggregations. (Table 1; for the complete list of identified species, see Supporting Information Table S3). Nearly three-quarters of the recorded insects belonged to Coleoptera (473; 33.6%) and Hymenoptera (572; 40.7%, of which 400 non-*Apis* and 144 honey bees) orders, followed by Diptera (167 hoverflies and 23 bee flies, 11.8% and 1.6% respectively) and Lepidoptera (147 butterflies and 25 moths, 10.4% and 1.8% respectively).

**Table 1.** Percentage of pictures allowing expert identification at family, genus and species level.

Taxonomic aggregations	Order	Family level (%)	Genus level (%)	Species level (%)	Total
Bee	Hymenoptera	98.5	97.2	57.9	544
Wasp	Hymenoptera	92.9	78.6	42.9	28
Bee fly	Diptera	100	91.3	21.7	23
Hoverfly	Diptera	100	95.8	68.3	167
Beetle	Coleoptera	97.7	85.8	53.9	473
Butterfly	Lepidoptera	100	93.2	85.0	147
Moth	Lepidoptera	96.0	96.0	76.0	25

Fifteen of identified bee species are classified as Data Deficient at European level (Nieto *et al*. 2014), while one hoverfly species, *Callicera spinolae* Rondani, 1844, is classified as Vulnerable. Some alien species were also spotted: the giant resin bee *Megachile sculpturalis* Smith, 1853, the butterfly *Cacyreus marshalli* Butler, 1898, and the Asian hornet *Vespa velutina* Lepeletier, 1836, an invasive species of Union Concern.

**Figure 6.**
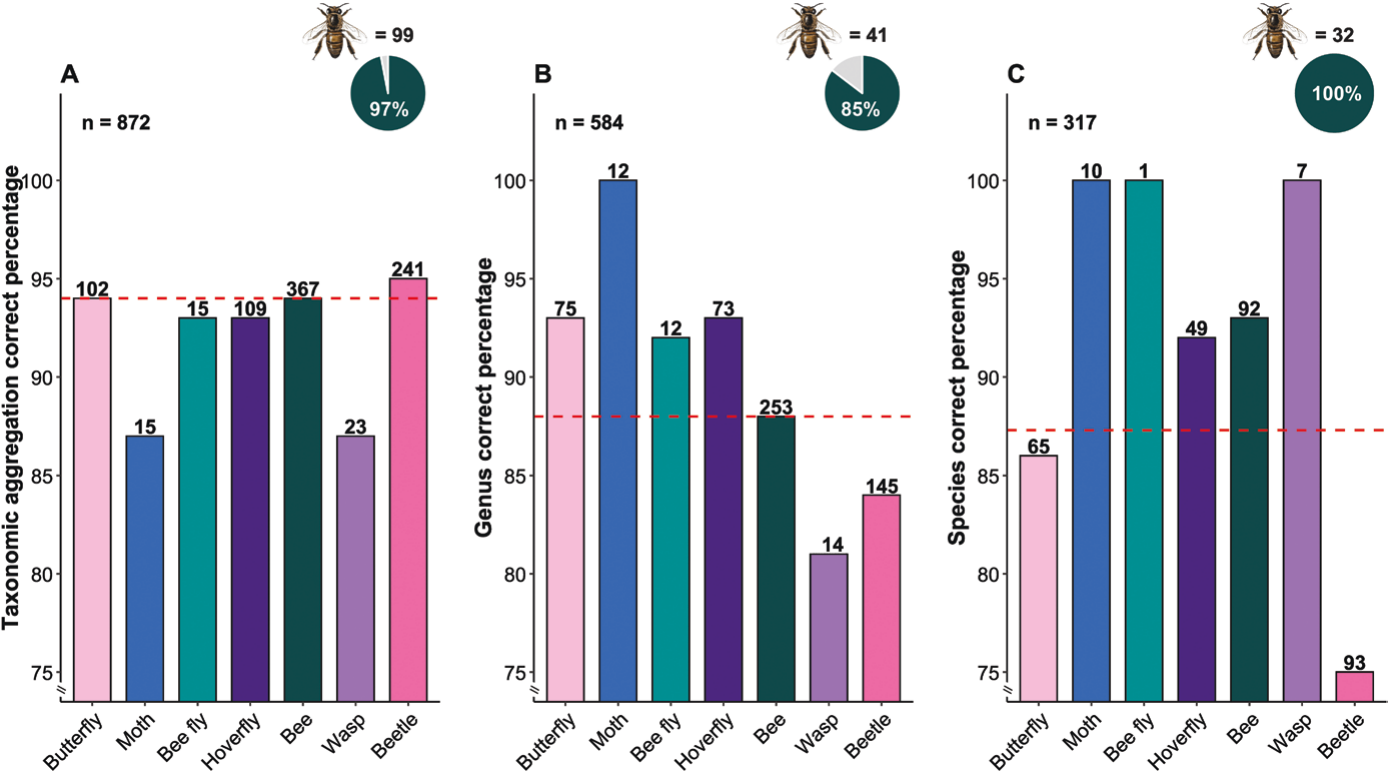
Percentage of insect photo records correctly identified at broad taxonomic aggregation (a), genus (b), and species level (c) by users, based on the answers to the question ‘What pollinator do you think it is?’ present in the submission page of the LIFE 4 Pollinators platform. The dashed lines indicate the average overall identification percentage. The pie charts in each graph specify the number of *Apis mellifera* records and percentage of correct identification. *Apis mellifera* illustration by Xavier Canyelles.

Users tried to attribute the record to one of the taxonomic aggregations suggested by the platform in 872 cases (62%), of which 94% were correctly identified (Fig. 6). Additional taxonomic details at the genus and species levels were provided for 584 and 317 records, respectively. Genus-level identification was accurate in 88% of cases, while 87% of insect photo records were correctly identified at the species level. As shown in Fig. 6, users’ accuracy was lower for wasps and moths at the functional group level (87%), for wasps at the genus level (81%), and for beetles at the species level (75%). In contrast, the honey bee was correctly identified at the species level in 100% of the observations where users provided the full binomial name. At the genus and broad taxonomic aggregation level, the identification accuracy for *Apis mellifera* was comparable.

Boschloo’s test shows a significant difference in the users’ identification correctness at the species level for beetle and a borderline significant difference for bees, while differences were not significant for the other functional groups (Supporting Information Table S2).

### Plants

**Figure 7.**
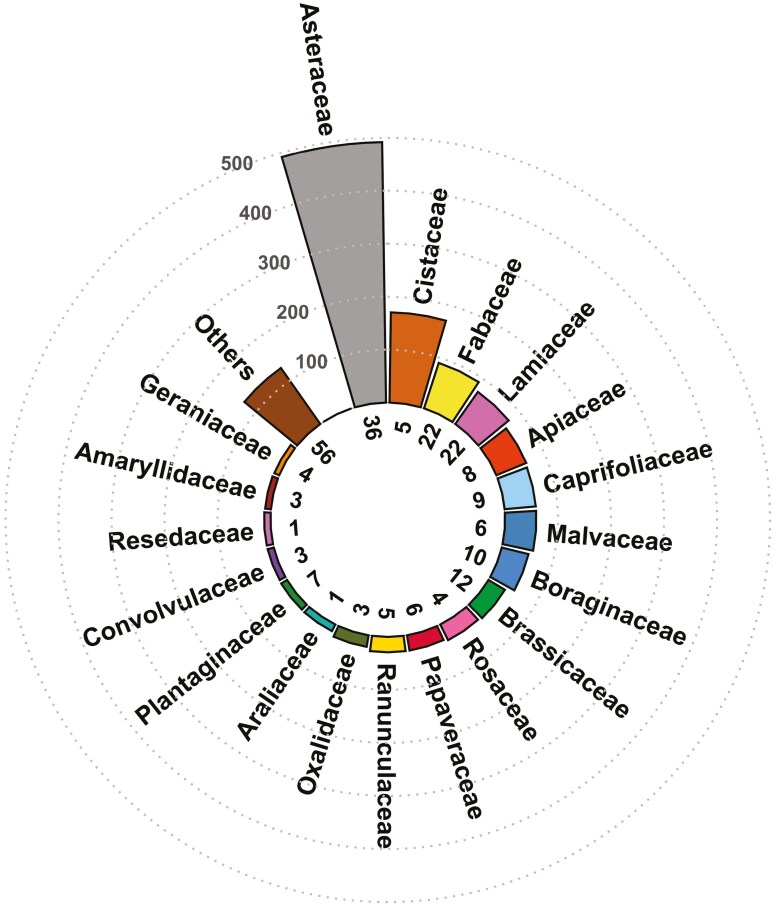
Number of photo records per plant family. The number of species is shown below each bar.

In total, 929 out of 1407 plant pictures (66.0%) were identified at the species level, 355 (25.2%) at the genus level, and the remaining 123 (8.7%) at the family level. More than half of the analysed records belonged to the families Asteraceae (35%), Cistaceae (12%), and Fabaceae (6%) (Fig. 7). The families with the highest number of spotted species were Asteraceae, with 36 species; Fabaceae and Lamiaceae, each with 22 species; and Brassicaceae, with 12 species (Supporting Information Table S3).

**Figure 8.**
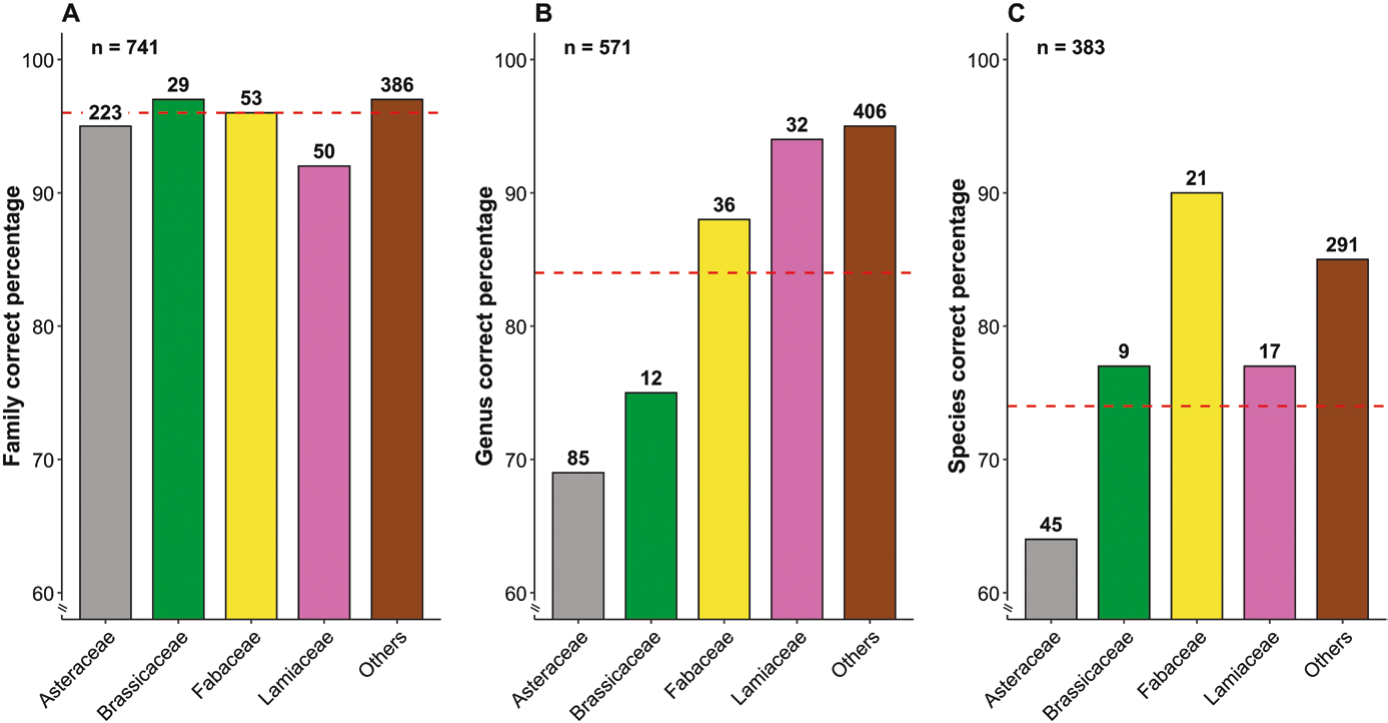
Percentage of plant photo records correctly identified at family (a), genus (b), and species level (c) by users, based on the answers to the question ‘What plant do you think it is?’ present in the submission page of the LIFE 4 pollinators platform. The dashed lines indicate the average overall identification percentage.

Among the plants identified at species level, two (*Olea europaea* L*., Vitex agnus-castus* L.) are classified as Data Deficient at European level (Bilz *et al*. 2011). Additionally, *Gymnadenia widderi* Teppner & E. Klein and *Kosteletzkya pentacarpos* (L.) Ledeb. are classified as Endangered and Vulnerable, respectively. Forty-nine species are alien to the observation areas, and *Oxalis pes-caprae* L. is invasive in Europe. Regarding the identifications provided by users (Fig. 8), 383 records reported the species name, 571 the genus, and 741 the family. Of these, 74%, 87%, and 96% were correct, respectively. The plant families significantly less recognized compared to the overall percentage of correct identifications are Asteraceae (69% at genus level, *P* < 0.001) and Oxalidaceae (18% at species level, *P* < 0.001). The latter, however, presents only 17 identifications by users (for information on all families, see Supplementary Material Table S4).

### Plant–pollinator interactions

**Figure 9.**
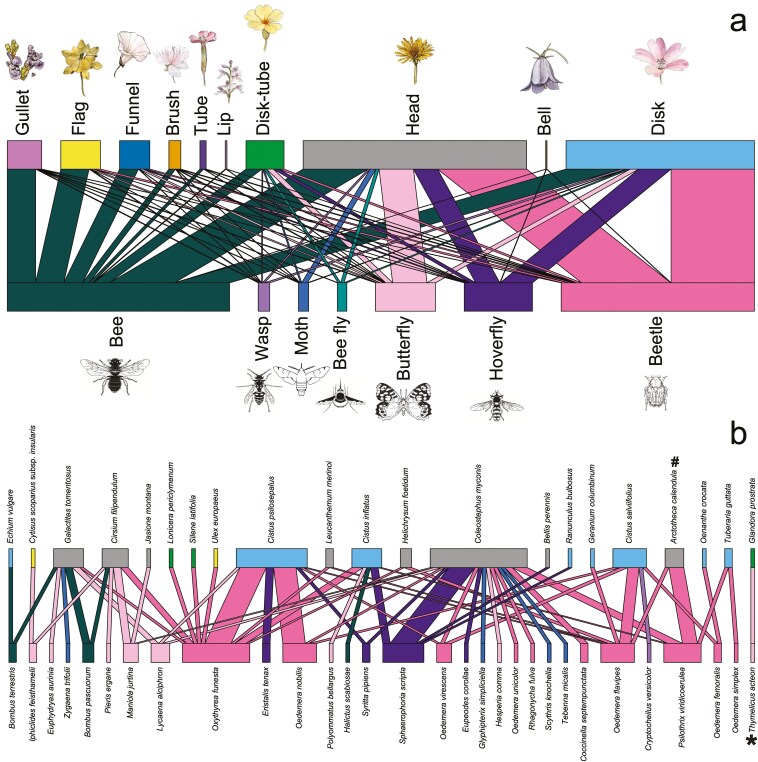
The network of floral shapes and visiting insects, derived from photo records on the LIFE 4 Pollinators platform (a), and alongside the network of pollinator-plant species from Ons Island (b), was obtained from a subset of photo records uploaded to the platform during a bioblitz conducted in May 2023. *#* indicates alien species, while * Near Threatened species. Insect illustration obtained from http://divulgare.net, plant illustrations by Marta Barberis.

The two most common interactions recorded (Fig. 9a) involve beetles with ‘Head’ (46%) and ‘Disk’ (43%) floral shape, both of which are considered generalist. Bees were recorded on nearly all floral shapes (39% of the total interactions). Like for beetles, the most common floral shape on which bees were recorded the most were the ‘Head’ (29%) and ‘Disk’ (25%) ones. Butterflies showed a strong association with the ‘Head’ floral shape (57%). ‘Disk’ - and ‘Head’ floral shapes were the ones where hoverflies were most frequently photographed (47% and 36%, respectively). Regarding bee flies, almost an equal number of records pictured an interaction with ‘Head’ (35%), ‘Disk’ (30%), and ‘Disk-Tube’ (26%) floral shape. Moths were mostly recorded on the ‘Head’ inflorescences (56%), while wasps on ‘Disk’ flowers (46%). The least represented floral shapes (0.5 %) were the ‘Lip’ (*n *= 4) and ‘Bell’ (*n *= 3) ones.

Focusing on the Ons Island dataset subset (Fig. 9b), interactions between 21 plant species and 30 pollinator species were spotted. Among the pollinators, the most represented functional group was that of beetles, with 10 species, followed by butterflies (8 species), and both hoverflies and moths (4 species each), whereas bees and wasps were represented by 3 and 1 species, respectively. Notably, the network included *Thymelicus acteon* Rottemburg, 1775, a butterfly classified as Near Threatened at the European level, and *Arctotheca calendula* (L.) K. Lewin, an alien plant in Europe.

## Discussion

The platform has proven valuable for collecting data on plants and pollinators, including IUCN Red List species, documenting plant-pollinator interactions across Natura 2000 areas and larger scales, and assessing citizen scientists’ ability to identify taxa from broad to specific levels.

One of the disadvantages of citizen science is the potential geographical bias, particularly if contributors are given a free choice in where to perform their observations (Gregory *et al*. 2005; Herrera 2019). In the current study, more than 90% of the photo records uploaded to the platform come from the project partners’ countries Spain, Italy, and Greece, where most of the activities were implemented. This result is clearly due to the numerous in-person events held in these countries, confirming the effectiveness of face-to-face events in engaging people in Citizen Science projects (Requier *et al*. 2020).

The taxonomic diversity of recorded pollinators demonstrates how the platform can be a valuable tool for expanding our knowledge of these insects, flanking and complementing existing tools focused on narrower taxonomic groups or different geographical regions (e.g. Prudic *et al*. 2017; Flaminio *et al*. 2021). Additionally, it provides data on Data Deficient species, including those that are threatened, alien, and invasive. However, as we did not require a minimum threshold of photograph quality, sometimes either low quality or lack of the taxonomic details needed for identification led to a relatively low number of determinations at the species level.

Unlike other citizen science projects, such as the Butterfly Monitoring Scheme, which rely on standardized methodologies (e.g. Pollard *et al*. 1994), volunteers contributing to our platform through photo uploads were not provided with a specific protocol. Consequently, this led to a bias in the types of pollinators recorded as well as in the taxa of flowers they were observed on—an expected side effect of non-standardized data collection. However, unlike initiatives such as the UK Pollinator Monitoring Scheme Fit-Count project (UK Pollinator Monitoring Scheme 2024), our platform offers citizen scientists the flexibility to select the plant species on which they record their observations. Moreover, every interaction is documented through photographs, enabling both a broader representation of plant species and a rigorous verification of pollinator and plant identifications by a team of taxonomists. This approach also facilitates, where possible, the identification of insects and plants at more precise taxonomic levels. For Beewatching, Flaminio *et al*. (2021) reported that the majority of bee species were those bigger in size. Similarly, the higher number of beetle records observed on the LIFE 4 Pollinators platform may be due to the greater ease of photographing them, since they have a more stationary behavior and visit flowers not only for nutritional rewards, but also for nesting and mating sites (Gottsberger 1990; Mayer *et al*. 2006), while for bees the high number may be due to the fact that they are the most popular pollinators (Ollerton 2021). Regarding the visited flowering plants, the greater abundance of Asteraceae, Cistaceae, and Fabaceae may be due to the greater attractiveness of these families for observers, given that they have relatively large and showy flowers.

The network of interactions between pollinator functional groups and floral shapes (*sensu*  Faegri and Van der Pijl 1979) would confirm the validity of floral syndromes concept (Dellinger 2020), emphasizing the ecological role of plants with generalist floral shapes (Head, Disk), that support all major pollinator functional groups. In accordance with the literature (Danforth 2007), our results show that, when taken altogether, bees are generalist visitors, being equally recorded on all different floral shapes. In fact, observers recorded bees equally across all floral shapes, likely due to the diverse floral diet and the related diversity of morpho-functional traits characterizing this insect taxon, in accordance with the literature (Danforth 2007).

The Ons network—which includes a threatened butterfly species and an alien plant species—demonstrates the importance of citizen science events (such as bioblitzes) carried out under the supervision of expert taxonomists. As data on plant-insect interactions are deficient for Natura 2000 sites, such information represents an important step for filling in the current gap.

Though qualitative, plant-pollinator networks obtained from data collected through citizen science initiatives may be useful in showcasing the direction that conservation measures should point, supporting a system-ecology approach focused on species interactions as the units to be conserved within larger ecosystems (Borchardt *et al*. 2021). The advantage is the possibility to extrapolate information and address specific investigations to plan interventions in areas where available information is suboptimal or missing, therefore allowing timely actions in a fast-changing environment. Given that the quality of data recorded by non-specialists within the framework of Citizen Science projects remains a topic of concern (Aceves-Bueno *et al*. 2017, and therein references), identification and validation by expert taxonomists yield more accurate data compared to other citizen science projects (such as iNaturalist platform, https://www.inaturalist.org), making the platform data more accurate for meta-analysis and data collection in urban and agricultural areas as well as Natura 2000 sites. Our study shows that users can correctly identify pollinators and plants with a high degree of accuracy at a high taxonomic level (broad taxonomic aggregations for pollinators, families for plants), while the percentage of correct identification decreases at the genus and species levels. This result aligns with the study by Falk *et al*. (2019), which analyzed data obtained by two Citizen Science projects to determine the ability of recorders to correctly identify bumblebee species. In their study, Falk *et al*. (2019) found that despite access to project-specific identification materials, the recorders’ ability was low. This result suggests that beyond the simplified tools available and freely downloadable from the LIFE 4 Pollinators project website, the implementation of trainings could increase taxonomic ability, while making users also aware of the fact that not all insect or plant species can be identifiable without inspecting samples under a stereomicroscope, due to the great diversity characterizing the Mediterranean area. Our results indicate that users are better able to recognize bee species than beetles. This can be attributed to the fact that bees are among the most well-known pollinators and one of the most represented in natural and agricultural ecosystems (Hung *et al*. 2018). Their prominence in education and media has made bees especially familiar to the general public, despite beetles being the largest and most diverse order of insects (Stork 2018). This complexity likely contributes to the lower accuracy in identifying beetle species. However, this result should be interpreted with caution, as over a quarter of our bee observations pertain to *Apis mellifera* Linnaeus, 1758, the honey bee, a species widely recognized by the general public, even at the species level. A similar result aligns with that reported by Flaminio *et al*. (2021) within the framework of the Beewatching project, for which such species appeared as the second most abundant taxon recorded (following the genus *Bombus*). The substantial presence of honey bees in our dataset also aligns with the widespread presence of managed hives across the Mediterranean region (Herrera 2020).

A low user retention has been observed, with most users uploading only a limited number of photos within a relatively short timeframe. This pattern is common in citizen science projects, where a subset of individuals, known as ‘super volunteers’, tend to contribute more consistently and provide higher-quality data (Sauermann and Franzoni 2015; Haklay 2016; Jennett *et al*. 2016). Research by See *et al*. (2016) highlights that providing timely feedback and effectively communicating the value of contributions are key strategies for enhancing user retention. Thus, improving the platform’s responsiveness to users, alongside transparent communication about data use, could positively impact engagement (Aspuru *et al*. 2016; Grossberndt and Liu 2016; Grainger 2017). Adding an interactive discussion blog or integrating the platform with social media could foster a sense of community, enabling users to quickly report issues and interact more effectively (Dickinson *et al*. 2012; Druschke and Seltzer 2012; Cooper *et al*. 2017). Additionally, implementing gamification elements—such as leaderboards, rewards, and user competitions—may further encourage engagement, particularly among younger users (Castell *et al*. 2015; Kotovirta *et al*. 2015; Gutiérrez *et al*. 2016). While anonymous photo uploads facilitate data collection, they also limit insights into the socio-economic profiles of users. Outlining users’ profiles may allow the planning of targeted activities for conservation purposes (Domroese and Johnson 2017). Other than increasing knowledge, a second critical output of the platform (and more in general, of any CS project) may be a change of attitude towards the studied group (Flaminio *et al*. 2021). People provided with information on conservation issues and engaged in CS projects are more likely to undertake conservation actions (Lewandowski and Oberhauser 2017).

Taken together, these strategies highlight pathways for strengthening user retention and maximizing the platform’s contribution to citizen science.

## Conclusions

The LIFE 4 Pollinators platform provides a dual benefit by advancing both scientific research and public engagement in biodiversity conservation. As a digital tool hosted on the project website, it enables systematic data collection to support scientific research, biodiversity management, and conservation strategies. With an accessible, anonymous data-entry format and comprehensive resources for plant and pollinator identification, the platform empowers citizen scientists to contribute observations on pollinator diversity across Mediterranean target areas and Natura 2000 sites (Fig. 9) while deepening their understanding of biodiversity in these focus regions. This engagement supplies policymakers and protected area managers with critical data to shape informed, targeted conservation strategies, while fostering a reconnection with nature and an awareness of biodiversity conservation and pollinator health (Domroese and Johnson 2017; Fraisl *et al*. 2022). Through active participation, individuals gain a more comprehensive understanding of ecological systems, making scientific processes more transparent and accessible. By enabling the public to contribute data and interact with conservation science, the LIFE 4 Pollinators platform, now over three years old, has successfully cultivated a community of citizen scientists and academic people invested in documenting pollinator and plant diversity. Looking forward, the platform—which will remain active for at least the next decade—holds substantial potential to support targeted research on priority or invasive species, generating insights into specific conservation challenges and guiding more adaptive, effective biodiversity protection strategies. Future research engaging a Citizen Science approach should also encourage targeted, small-scale investigation initiatives allowing for a more comprehensive understanding of pollinator status and local trends (see Herrera 2019). By improving data coverage in underrepresented regions and habitats, this information will enhance the robustness of pollinator conservation policies and contribute to a more comprehensive understanding of pollinator trends over time across diverse ecological landscapes.

## Supplementary Material

plaf023_suppl_Supplementary_Materials

## Data Availability

All data can be found in the Zenodo repository with DOI 10.5281/zenodo.15045829
