# Supplementary material for: LIFE 4 Pollinators’ platform: How citizen science can help monitoring plants and pollinators
Source: AoB Plants. 2025 Apr 18;17(3):plaf023. doi: 10.1093/aobpla/plaf023 (PMC12190796; doi:10.1093/aobpla/plaf023)
Supplement: plaf023_suppl_Supplementary_Materials [file plaf023_suppl_supplementary_materials.docx]

**SUPPORTING INFORMATION for “LIFE4Pollinators’ Platform: How Citizen Science Can Help Monitoring Plants and Pollinators”**

**Table S1.** Observations in Natura 2000 sites. The table displeys the country, site code, site name, site type, and the number of observations. Sites are listed in descending order based on the number of observations. Site types are categorised as follows: A = protected under the Birds Directive (SPA sites); B = protected under the Habitats Directive (pSCI, SCI and SAC sites); C = protected under both the Birds and Habitats Directives.

| **Country** | **Site code** | **Site name** | **Site type** | **No. of records** |
| --- | --- | --- | --- | --- |
| Spain | ES0000254/ES1140004 | Illa de Ons/Complexo Ons - O Grove | A/B | 527 |
| Spain | ES0000001 | Illas Cíes | C | 225 |
| Greece | GR2520006 | Oros Parnonas (Kai Periochi Malevis) | B | 46 |
| Greece | GR4110010/GR4110003 | Notiodytiki Chersonisos, Apolithomeno Dasos Lesvou/Lesvos: Dytiki Chersonisos - Apolithomeno Dasos | A/B | 27 |
| Greece | GR1270005 | Oros Stratonikon - Koryfi Skamni | B | 26 |
| Italy | IT3250003 | Penisola del Cavallino: biotopi litoranei | C | 16 |
| Spain | ES1110006 | Complexo húmido de Corrubedo | B | 13 |
| Italy | IT7110128/  IT7110202 | Parco Nazionale Gran Sasso - Monti della Laga/Gran Sasso | A/B | 7 |
| Italy | IT20B0401/  IT20A0004 | Parco Regionale Oglio Sud/Le Bine | A/B | 7 |
| Italy | ITA070029/  ITA070001 | Biviere di Lentini, tratto mediano e foce del Fiume Simeto e area antistante la foce/Foce del Fiume Simeto e Lago Gornalunga | A/B | 6 |
| Greece | GR4110013/GR4110005 | Lesvos: Kolpos Geras, Eli Ntipi Kai Charamida/Lesvos Kolpos Geras, Elos Ntipi Kai Oros Olympos – Potamos Evergetoulas | A/B | 6 |
| Italy | IT51A0008 | Monte d'Alma | B | 5 |
| Italy | IT4050001 | Gessi Bolognesi, Calanchi dell'Abbadessa | C | 5 |
| Spain | ES0000145 | Mondragó | C | 5 |
| Spain | ES0000471/ES0000023 | l'Albufera (ZEPA)/L'Albufera | A/B | 3 |
| Italy | IT5310027/  IT5310013 | Mombaroccio e Beato Sante/Mombaroccio | A/B | 3 |
| Spain | ES0000437/ES1130007 | Pena Trevinca/Pena Trevinca | A/B | 3 |
| Greece | GR4220016 | Nisos Paros: petaloudes | B | 3 |
| Italy | IT4050012 | Contrafforte Pliocenico | C | 3 |
| Italy | IT5310029/IT5310016 | Furlo/Gola del Furlo | A/B | 2 |
| Spain | ES0000544/ES5310095 | Son Real/Can Picafort | A/B | 2 |
| Greece | GR4110005 | Lesvos Kolpos Geras, Elos Ntipi Kai Oros Olympos – Potamos Evergetoulas | B | 2 |
| Italy | IT5140008 | Monte Morello | B | 2 |
| Italy | IT4050029 | Boschi di San Luca e Destra Reno | C | 2 |
| Italy | IT4050002 | Corno alle Scale | C | 2 |
| Italy | IT2080301/  IT2010014 | Boschi del Ticino/Turbigaccio, Boschi di Castelletto e Lanca di Bernate | A/B | 1 |
| Italy | IT5180004/  IT5180003 | Camaldoli, Scodella, Campigna, Badia Prataglia/Giogo Seccheta | A/B | 1 |
| Italy | IT6030085 | Comprensorio Bracciano-Martignano | A | 1 |
| Greece | GR2230008 | Diapontia Nisia (Othonoi, Ereikousa, Mathraki Kai Vrachonisides) | A | 1 |
| Spain | ES0000499 | Espacio marino de las Rías Baixas de Galicia | A | 1 |
| Spain | ES0000520/ES5310005 | Espacio marino del norte de Mallorca/Badies de Pollença i Alcúdia | A/B | 1 |
| Slovenia | SI5000023/  SI3000276 | Kras/Kras | A/B | 1 |
| Greece | GR4210034 | Nisides Voreion Dodekanison Kai Thalassia Periochi | A | 1 |
| Italy | IT7120132/  IT7212121 | Parco Nazionale d'Abruzzo, Lazio e Molise ed aree limitrofe/Gruppo della Meta - Catena delle Mainarde | A/B | 1 |
| Italy | IT7120132 | Parco Nazionale d'Abruzzo, Lazio e Molise ed aree limitrofe | A | 1 |
| Italy | IT7140129/  IT7140203 | Parco Nazionale della Maiella/Maiella | A/B | 1 |
| Italy | ITB013012/  ITB010002 | Stagno di Pilo, Casaraccio e Saline di Stintino/Stagno di Pilo e di Casaraccio | A/B | 1 |
| Italy | IT4050016 | Abbazia di Monteveglio | B | 1 |
| Spain | ES1120001 | Ancares - Courel | B | 1 |
| Italy | IT3120017 | Campobrun | B | 1 |
| Italy | ITB042216 | Capo di Pula | B | 1 |
| Italy | IT5180002 | Foreste Alto Bacino dell'Arno | B | 1 |
| Italy | IT5180018 | Foreste di Camaldoli e Badia Prataglia | B | 1 |
| Greece | GR1260001 | Limni Kerkini - Krousia - Koryfes Orous Beles, Angistro - Charopo | B | 1 |
| Spain | ES1120010 | Negueira | B | 1 |
| Italy | IT1110057 | Serra di Ivrea | B | 1 |
| Italy | ITB040023 | Stagno di Cagliari, Saline di Macchiareddu, Laguna di Santa Gilla | B | 1 |
| Italy | ITB010003 | Stagno e ginepreto di Platamona | B | 1 |
| Italy | IT5140012 | Vallombrosa e Bosco di S. Antonio | B | 1 |
| Italy | IT1160056 | Alpi Marittime | C | 1 |
| Italy | IT5310022 | Fiume Metauro da Piano di Zucca alla foce | C | 1 |
| Spain | ES0000079 | La Victòria | C | 1 |
| Italy | IT4050032 | Monte dei Cucchi, Pian di Balestra | C | 1 |
| Italy | IT4080003 | Monte Gemelli, Monte Guffone | C | 1 |
| Italy | ITB021103 | Monti del Gennargentu | C | 1 |

**Table S2.** Photo records of insects not belonging to the focal functional groups of the platform (*n* = 143), by Order.

| **Order** | **No. of photo records** | **Percentage** |
| --- | --- | --- |
| Araneae | 24 | 16.8% |
| Diptera ^a^ | 80 | 55.9% |
| Hemiptera | 20 | 14.0% |
| Hymenoptera ^b^ | 7 | 4.9% |
| Mecoptera | 1 | 0.7% |
| Neuroptera | 4 | 2.8% |
| Orthoptera | 7 | 4.9% |

^a^ Other than hoverflies and bee flies.

^b^ Other than bees and wasps.

**Table S3.** List of identified pollinators species. The table presents the following information: Order, Functional group, Family, species binomial, IUCN Red List classification at the European level, ASA = Alien Status Assessment, ASAS=Alien Status Assessment Source, PPT=Primary Pathway Type as listed in the EASIN database, ISA=Invasive Status Assessment in Europe, and ISES= source of the Invasive Status Evaluation. Identifications at the genus level are provided only when a single occurrence is recorded. All alien and invasive species are classified as such throughout Europe.

| Order | Functional group | Family | *Species* | IUCN EU | A S A | A S A S | P P T | I S A | I S E S |
| --- | --- | --- | --- | --- | --- | --- | --- | --- | --- |
| Coleoptera | Beetle | Buprestidae | *Anthaxia hungarica* Scopoli, 1772 | - | - | - | - | - | - |
| Coleoptera | Beetle | Cantharidae | *Rhagonycha fulva* Scopoli, 1763 | - | - | - | - | - | - |
| Coleoptera | Beetle | Cerambycidae | *Certallum ebulinum* Linnaeus, 1767 | - | - | - | - | - | - |
| Coleoptera | Beetle | Cerambycidae | *Plagionotus floralis* Pallas, 1773 | - | - | - | - | - | - |
| Coleoptera | Beetle | Cerambycidae | *Stenurella bifasciata* O. F. Müller, 1776 | - | - | - | - | - | - |
| Coleoptera | Beetle | Chrysomelidae | *Chrysolina americana* Linnaeus, 1758 | - | - | - | - | - | - |
| Coleoptera | Beetle | Chrysomelidae | *Cryptocephalus rugicollis* Olivier, 1792 | - | - | - | - | - | - |
| Coleoptera | Beetle | Chrysomelidae | *Cryptocephalus vittatus* Fabricius, 1775 | - | - | - | - | - | - |
| Coleoptera | Beetle | Chrysomelidae | *Exosoma lusitanicum* Linnaeus, 1767 | - | - | - | - | - | - |
| Coleoptera | Beetle | Cleroidea | *Trichodes alvearius* Fabricius, 1792 | - | - | - | - | - | - |
| Coleoptera | Beetle | Cleroidea | *Trichodes punctatus* Fischer von Waldheim, 1829 | - | - | - | - | - | - |
| Coleoptera | Beetle | Coccinellidae | *Adalia decempunctata* Linnaeus, 1758 | - | - | - | - | - | - |
| Coleoptera | Beetle | Coccinellidae | *Coccinella septempunctata* Linnaeus, 1758 | - | - | - | - | - | - |
| Coleoptera | Beetle | Coccinellidae | *Hippodamia variegata* Goeze, 1777 | - | - | - | - | - | - |
| Coleoptera | Beetle | Glaphyridae | *Eulasia pareyssei* Brullé, 1832 | - | - | - | - | - | - |
| Coleoptera | Beetle | Glaphyridae | *Pygopleurus foina* Reitter, 1890 | - | - | - | - | - | - |
| Coleoptera | Beetle | Meloidae | *Mylabris quadripunctata* Linnaeus, 1767 | - | - | - | - | - | - |
| Coleoptera | Beetle | Melyridae | *Attalus cyaneus* Fabricius, 1792 | - | - | - | - | - | - |
| Coleoptera | Beetle | Melyridae | *Psilothrix viridicoerulea* Geoffroy, 1785 | - | - | - | - | - | - |
| Coleoptera | Beetle | Miridae | *Calocoris nemoralis* Fabricius, 1787 | - | - | - | - | - | - |
| Coleoptera | Beetle | Oedemeridae | *Oedemera barbara* Fabricius, 1792 | - | - | - | - | - | - |
| Coleoptera | Beetle | Oedemeridae | *Oedemera croceicollis* Gyllenhal, 1827 | - | - | - | - | - | - |
| Coleoptera | Beetle | Oedemeridae | *Oedemera femoralis* Olivier, 1803 | LC | - | - | - | - | - |
| Coleoptera | Beetle | Oedemeridae | *Oedemera flavipes* Fabricius, 1792 | LC | - | - | - | - | - |
| Coleoptera | Beetle | Oedemeridae | *Oedemera lurida* Marsham, 1802 | - | - | - | - | - | - |
| Coleoptera | Beetle | Oedemeridae | *Oedemera nobilis* Scopoli, 1763 | - | - | - | - | - | - |
| Coleoptera | Beetle | Oedemeridae | *Oedemera podagrariae* Linnaeus, 1767 | - | - | - | - | - | - |
| Coleoptera | Beetle | Oedemeridae | *Oedemera pthysica* Scopoli, 1763 | - | - | - | - | - | - |
| Coleoptera | Beetle | Oedemeridae | *Oedemera rufofemorata* Germar, 1824 | - | - | - | - | - | - |
| Coleoptera | Beetle | Oedemeridae | *Oedemera simplex* Linnaeus, 1767 | - | - | - | - | - | - |
| Coleoptera | Beetle | Oedemeridae | *Oedemera unicolor* W. Schmidt, 1846 | - | - | - | - | - | - |
| Coleoptera | Beetle | Oedemeridae | *Oedemera virescens* Linnaeus, 1767 | - | - | - | - | - | - |
| Coleoptera | Beetle | Pyrrhocoridae | *Pyrrhocoris apterus* Linnaeus, 1758 | - | - | - | - | - | - |
| Coleoptera | Beetle | Scarabaeidae | *Cetonia aurata* Linnaeus, 1758 | - | - | - | - | - | - |
| Coleoptera | Beetle | Scarabaeidae | *Chasmatopterus* villosulus Illiger, 1803 | - | - | - | - | - | - |
| Coleoptera | Beetle | Scarabaeidae | *Oxythyrea cinctella* Schaum, 1841 | - | - | - | - | - | - |
| Coleoptera | Beetle | Scarabaeidae | *Oxythyrea funesta* Poda, 1761 | - | - | - | - | - | - |
| Coleoptera | Beetle | Scarabaeidae | *Phyllopertha horticola* Linnaeus, 1758 | - | - | - | - | - | - |
| Coleoptera | Beetle | Scarabaeidae | *Tropinota hirta* Poda, 1761 | - | - | - | - | - | - |
| Coleoptera | Beetle | Scarabaeidae | *Valgus hemipterus* Linnaeus, 1758 | LC | - | - | - | - | - |
| Diptera | Bee fly | Bombyliidae | *Bombylius trichurus* Pallas, 1818 | - | - | - | - | - | - |
| Diptera | Bee fly | Bombyliidae | *Exoprosopa minois* Loew, 1869 | - | - | - | - | - | - |
| Diptera | Bee fly | Bombyliidae | *Exoprosopa pandora* Fabricius, 1805 | - | - | - | - | - | - |
| Diptera | Bee fly | Bombyliidae | *Lomatia* sp. | - | NA | - | - | - | - |
| Diptera | Bee fly | Bombyliidae | *Usia versicolor* Fabricius, 1787 | - | - | - | - | - | - |
| Diptera | Hoverfly | Syrphidae | *Callicera spinolae* Rondani, 1844 | VU | - | - | - | - | - |
| Diptera | Hoverfly | Syrphidae | *Chrysotoxum intermedium* Meigen, 1822 | LC | - | - | - | - | - |
| Diptera | Hoverfly | Syrphidae | *Epistrophe eligans* Harris, 1780 | LC | - | - | - | - | - |
| Diptera | Hoverfly | Syrphidae | *Episyrphus balteatus* De Geer, 1776 | LC | - | - | - | - | - |
| Diptera | Hoverfly | Syrphidae | *Eristalinus aeneus* Scopoli, 1763 | LC | - | - | - | - | - |
| Diptera | Hoverfly | Syrphidae | *Eristalinus megacephalus* Rossi, 1794 | LC | - | - | - | - | - |
| Diptera | Hoverfly | Syrphidae | *Eristalinus taeniops* Wiedemann, 1818 | LC | - | - | - | - | - |
| Diptera | Hoverfly | Syrphidae | *Eristalis arbustorum* Linnaeus, 1758 | LC | - | - | - | - | - |
| Diptera | Hoverfly | Syrphidae | *Eristalis pertinax* Scopoli, 1763 | LC | - | - | - | - | - |
| Diptera | Hoverfly | Syrphidae | *Eristalis tenax* Linnaeus, 1758 | LC | - | - | - | - | - |
| Diptera | Hoverfly | Syrphidae | *Eupeodes corollae* Fabricius, 1794 | LC | - | - | - | - | - |
| Diptera | Hoverfly | Syrphidae | *Meliscaeva auricollis* Meigen, 1822 | LC | - | - | - | - | - |
| Diptera | Hoverfly | Syrphidae | *Merodon clavipes* Fallen, 1817 | LC | - | - | - | - | - |
| Diptera | Hoverfly | Syrphidae | *Merodon rufus* Meigen, 1838 | LC | - | - | - | - | - |
| Diptera | Hoverfly | Syrphidae | *Myathropa florea* Linnaeus, 1758 | LC | - | - | - | - | - |
| Diptera | Hoverfly | Syrphidae | *Scaeva pyrastri* Linnaeus, 1758 | LC | - | - | - | - | - |
| Diptera | Hoverfly | Syrphidae | *Sphaerophoria rueppelli* Wiedemann, 1820 | LC | - | - | - | - | - |
| Diptera | Hoverfly | Syrphidae | *Sphaerophoria scripta* Linnaeus, 1758 | LC | - | - | - | - | - |
| Diptera | Hoverfly | Syrphidae | *Syritta pipiens* Linnaeus, 1758 | LC | - | - | - | - | - |
| Diptera | Hoverfly | Syrphidae | *Syrphus ribesii* Linnaeus, 1758 | LC | - | - | - | - | - |
| Diptera | Hoverfly | Syrphidae | *Volucella zonaria* Poda, 1761 | LC | - | - | - | - | - |
| Hymenoptera | Bee | Apidae | *Amegilla albigena* Lepeletier, 1841 | LC | - | - | - | - | - |
| Hymenoptera | Bee | Apidae | *Anthophora agama* Radoszkowski, 1869 | DD | - | - | - | - | - |
| Hymenoptera | Bee | Apidae | *Anthophora bimaculata* Panzer, 1798 | LC | - | - | - | - | - |
| Hymenoptera | Bee | Apidae | *Anthophora crinipes* Smith, 1854 | DD | - | - | - | - | - |
| Hymenoptera | Bee | Apidae | *Anthophora dispar* Lepeletier, 1841 | LC | - | - | - | - | - |
| Hymenoptera | Bee | Apidae | *Anthophora plumipes* Pallas, 1772 | LC | - | - | - | - | - |
| Hymenoptera | Bee | Apidae | *Anthophora rubricrus* Dours, 1869 | DD | - | - | - | - | - |
| Hymenoptera | Bee | Apidae | *Apis mellifera* Linnaeus, 1758 | DD | - | - | - | - | - |
| Hymenoptera | Bee | Apidae | *Bombus argillaceus* Scopoli, 1763 | LC | - | - | - | - | - |
| Hymenoptera | Bee | Apidae | *Bombus hortorum* Linnaeus, 1761 | LC | - | - | - | - | - |
| Hymenoptera | Bee | Apidae | *Bombus pascuorum* Scopoli, 1763 | LC | - | - | - | - | - |
| Hymenoptera | Bee | Apidae | *Bombus pratorum* Linnaeus, 1761 | LC | - | - | - | - | - |
| Hymenoptera | Bee | Apidae | *Bombus ruderatus* Fabricius, 1775 | LC | - | - | - | - | - |
| Hymenoptera | Bee | Apidae | *Bombus sichelii* Radoszkowski, 1860 | LC | - | - | - | - | - |
| Hymenoptera | Bee | Apidae | *Bombus terrestris* Linnaeus, 1758 | LC | - | - | - | - | - |
| Hymenoptera | Bee | Apidae | *Bombus terrestris subsp. dalmatinus* Dalla Torre, 1882 | LC | NA | - | - | - | - |
| Hymenoptera | Bee | Apidae | *Ceratina cucurbitina* Rossi, 1792 | LC | - | - | - | - | - |
| Hymenoptera | Bee | Apidae | *Ceratina cyanea* Kirby, 1802 | LC | - | - | - | - | - |
| Hymenoptera | Bee | Apidae | *Ceratina parvula* Smith, 1854 | LC | - | - | - | - | - |
| Hymenoptera | Bee | Apidae | *Eucera longicornis* Linnaeus, 1758 | LC | - | - | - | - | - |
| Hymenoptera | Bee | Apidae | *Habropoda tarsata* Spinola, 1838 | LC | - | - | - | - | - |
| Hymenoptera | Bee | Apidae | *Xylocopa iris* Christ, 1791 | LC | - | - | - | - | - |
| Hymenoptera | Bee | Apidae | *Xylocopa pubescens* Spinola, 1838 | - | - | - | - | - | - |
| Hymenoptera | Bee | Apidae | *Xylocopa valga* Gerstäcker, 1872 | LC | - | - | - | - | - |
| Hymenoptera | Bee | Apidae | *Xylocopa violacea* Linnaeus, 1758 | LC | - | - | - | - | - |
| Hymenoptera | Bee | Colletidae | *Colletes succinctus* Linnaeus, 1758 | NT | - | - | - | - | - |
| Hymenoptera | Bee | Colletidae | *Hylaeus punctatus* Brullé, 1832 | LC | - | - | - | - | - |
| Hymenoptera | Bee | Halictidae | *Andrena crecca* Warncke, 1965 | - | - | - | - | - | - |
| Hymenoptera | Bee | Halictidae | *Andrena dorsata* Kirby, 1802 | DD | - | - | - | - | - |
| Hymenoptera | Bee | Halictidae | *Andrena flavipes* Panzer, 1799 | LC | - | - | - | - | - |
| Hymenoptera | Bee | Halictidae | *Andrena fulvitarsis* Brullé, 1832 | LC | - | - | - | - | - |
| Hymenoptera | Bee | Halictidae | *Andrena gravida* Imhoff, 1832 | DD | - | - | - | - | - |
| Hymenoptera | Bee | Halictidae | *Andrena morio* Brullé, 1832 | DD | - | - | - | - | - |
| Hymenoptera | Bee | Halictidae | *Andrena nigroaenea* Kirby, 1802 | LC | - | - | - | - | - |
| Hymenoptera | Bee | Halictidae | *Andrena scita* Eversmann, 1852 | DD | - | - | - | - | - |
| Hymenoptera | Bee | Halictidae | *Andrena thoracica* Fabricius, 1775 | DD | - | - | - | - | - |
| Hymenoptera | Bee | Halictidae | *Halictus cephalicus* Morawitz, 1873 | LC | - | - | - | - | - |
| Hymenoptera | Bee | Halictidae | *Halictus pollinosus* Sichel, 1860 | LC | - | - | - | - | - |
| Hymenoptera | Bee | Halictidae | *Halictus scabiosae* Rossi, 1790 | LC | - | - | - | - | - |
| Hymenoptera | Bee | Halictidae | *Lasioglossum malachurum* Kirby, 1802 | LC | - | - | - | - | - |
| Hymenoptera | Bee | Halictidae | *Lasioglossum nitidulum* Fabricius, 1804 | LC | - | - | - | - | - |
| Hymenoptera | Bee | Halictidae | *Lasioglossum villosulum* Kirby, 1802 | LC | - | - | - | - | - |
| Hymenoptera | Bee | Halictidae | *Nomioides facilis* Smith, 1853 | LC | - | - | - | - | - |
| Hymenoptera | Bee | Megachilidae | *Anthidiellum strigatum* Panzer, 1805 | LC | - | - | - | - | - |
| Hymenoptera | Bee | Megachilidae | *Anthidium florentinum* Fabricius,1775 | LC | - | - | - | - | - |
| Hymenoptera | Bee | Megachilidae | *Dioxys cinctus* Jurine, 1807 | LC | - | - | - | - | - |
| Hymenoptera | Bee | Megachilidae | *Eoanthidium insulare* Morawitz, 1874 | LC | - | - | - | - | - |
| Hymenoptera | Bee | Megachilidae | *Hoplitis tigrina* Morawitz 1872 | DD | - | - | - | - | - |
| Hymenoptera | Bee | Megachilidae | *Megachile lefebvrei* Lepeletier, 1841 | DD | - | - | - | - | - |
| Hymenoptera | Bee | Megachilidae | *Megachile parietina* Geoffroy, 1785 | LC | - | - | - | - | - |
| Hymenoptera | Bee | Megachilidae | *Megachile pilidens* Alfken, 1924 | LC | - | - | - | - | - |
| Hymenoptera | Bee | Megachilidae | *Megachile rotundata* Fabricius, 1787 | DD | - | - | - | - | - |
| Hymenoptera | Bee | Megachilidae | *Megachile sculpturalis* Smith, 1853 | - | YES | a | TR | - | - |
| Hymenoptera | Bee | Megachilidae | *Megachile sicula* Rossi, 1792 | DD | - | - | - | - | - |
| Hymenoptera | Bee | Megachilidae | *Osmia bicornis* Linnaeus, 1758 | LC | - | - | - | - | - |
| Hymenoptera | Bee | Megachilidae | *Osmia cornuta* Latreille, 1805 | LC | - | - | - | - | - |
| Hymenoptera | Bee | Megachilidae | *Osmia elegans* Tkalcu, 1992 | DD | - | - | - | - | - |
| Hymenoptera | Bee | Megachilidae | *Rhodanthidium septemdentatum* Latreille, 1809 | DD | - | - | - | - | - |
| Hymenoptera | Bee | Megachilidae | *Rhodanthidium sticticum* Fabricius, 1787 | DD | - | - | - | - | - |
| Hymenoptera | Wasp | Pompilidae | *Cryptocheilus versicolor* Scopoli, 1763 | - | - | - | - | - | - |
| Hymenoptera | Wasp | Sapygidae | *Sapyga quinquepunctata* Fabricius, 1781 | - | - | - | - | - | - |
| Hymenoptera | Wasp | Scoliidae | *Campsomeriella thoracica* Fabricius, 1787 | - | - | - | - | - | - |
| Hymenoptera | Wasp | Scoliidae | *Dasyscolia ciliata* Fabricius, 1787 | - | - | - | - | - | - |
| Hymenoptera | Wasp | Scoliidae | *Megascolia maculata* Drury, 1773 | - | - | - | - | - | - |
| Hymenoptera | Wasp | Scoliidae | *Scolia hirta* Schrank, 1781 | - | - | - | - | - | - |
| Hymenoptera | Wasp | Scoliidae | *Scolia maculata flavifrons* Fabricius, 1775 | - | - | - | - | - | - |
| Hymenoptera | Wasp | Vespidae | *Polistes gallicus* Linnaeus, 1767 | - | - | - | - | - | - |
| Hymenoptera | Wasp | Vespidae | *Vespa orientalis* Linnaeus, 1771 | - | - | - | - | - | - |
| Hymenoptera | Wasp | Vespidae | *Vespa velutina nigrithorax* Lepeletier, 1836 | - | YES | a,b | TR | Yes | a,b |
| Lepidoptera | Butterfly | Adelidae | *Adela croesella* Scopoli, 1763 | - | - | - | - | - | - |
| Lepidoptera | Butterfly | Choreutidae | *Anthophila fabriciana* Linnaeus, 1767 | - | - | - | - | - | - |
| Lepidoptera | Butterfly | Choreutidae | *Tebenna micalis* Mann, 1857 | - | - | - | - | - | - |
| Lepidoptera | Butterfly | Hesperiidae | *Carcharodus alceae* Esper, 1780 | LC | - | - | - | - | - |
| Lepidoptera | Butterfly | Hesperiidae | *Hesperia comma* Linnaeus, 1758 | LC | - | - | - | - | - |
| Lepidoptera | Butterfly | Hesperiidae | *Ochlodes venata* subsp. *faunus* Turati, 1905 | - | - | - | - | - | - |
| Lepidoptera | Butterfly | Hesperiidae | *Thymelicus acteon* Rottemburg, 1775 | NT | - | - | - | - | - |
| Lepidoptera | Butterfly | Hesperiidae | *Thymelicus sylvestris* Poda, 1761 | LC | - | - | - | - | - |
| Lepidoptera | Butterfly | Lycaenidae | *Aricia agestis* Denis & Schiffermüller, 1775 | LC | - | - | - | - | - |
| Lepidoptera | Butterfly | Lycaenidae | *Cacyreus marshalli* Butler, 1898 | - | YES | a | TR | - | - |
| Lepidoptera | Butterfly | Lycaenidae | *Celastrina argiolus* Linnaeus, 1758 | LC | - | - | - | - | - |
| Lepidoptera | Butterfly | Lycaenidae | *Leptotes pirithous* Linnaeus, 1767 | LC | - | - | - | - | - |
| Lepidoptera | Butterfly | Lycaenidae | *Lycaena alciphron* Rottemburg, 1775 | LC | - | - | - | - | - |
| Lepidoptera | Butterfly | Lycaenidae | *Lycaena phlaeas* Linnaeus, 1761 | LC | - | - | - | - | - |
| Lepidoptera | Butterfly | Lycaenidae | *Lysandra bellargus* Rottemburg, 1775 | LC | - | - | - | - | - |
| Lepidoptera | Butterfly | Lycaenidae | *Polyommatus icarus* Rottemburg, 1775 | LC | - | - | - | - | - |
| Lepidoptera | Butterfly | Nymphalidae | *Argynnis paphia* Linnaeus, 1758 | LC | - | - | - | - | - |
| Lepidoptera | Butterfly | Nymphalidae | *Coenonympha pamphilus* Linnaeus, 1758 | LC | - | - | - | - | - |
| Lepidoptera | Butterfly | Nymphalidae | *Euphydryas aurinia* Rottemburg, 1775 | LC | - | - | - | - | - |
| Lepidoptera | Butterfly | Nymphalidae | *Hipparchia algirica* Oberthür, 1876 | LC | - | - | - | - | - |
| Lepidoptera | Butterfly | Nymphalidae | *Issoria lathonia* Linnaeus, 1758 | LC | - | - | - | - | - |
| Lepidoptera | Butterfly | Nymphalidae | *Maniola jurtina* Linnaeus, 1758 | LC | - | - | - | - | - |
| Lepidoptera | Butterfly | Nymphalidae | *Melanargia galathea* Linnaeus, 1758 | LC | - | - | - | - | - |
| Lepidoptera | Butterfly | Nymphalidae | *Melanargia larissa* Geyer, 1828 | LC | - | - | - | - | - |
| Lepidoptera | Butterfly | Nymphalidae | *Melitaea athalia* Rottemburg, 1775 | LC | - | - | - | - | - |
| Lepidoptera | Butterfly | Nymphalidae | *Melitaea phoebe* Denis & Schiffermüller, 1775 | LC | - | - | - | - | - |
| Lepidoptera | Butterfly | Nymphalidae | *Pararge aegeria* Linnaeus, 1758 | LC | - | - | - | - | - |
| Lepidoptera | Butterfly | Nymphalidae | *Vanessa atalanta* Linnaeus, 1758 | LC | - | - | - | - | - |
| Lepidoptera | Butterfly | Nymphalidae | *Vanessa cardui* Linnaeus, 1758 | LC | - | - | - | - | - |
| Lepidoptera | Butterfly | Papilionidae | *Iphiclides feisthamelii* Duponchel, 1832 | - | - | - | - | - | - |
| Lepidoptera | Butterfly | Papilionidae | *Iphiclides podalirius* Linnaeus, 1758 | LC | - | - | - | - | - |
| Lepidoptera | Butterfly | Papilionidae | *Papilio machaon* Linnaeus, 1758 | LC | - | - | - | - | - |
| Lepidoptera | Butterfly | Papilionidae | *Parnassius apollo* Linnaeus, 1758 | LC | - | - | - | - | - |
| Lepidoptera | Butterfly | Papilionidae | *Zerynthia rumina* Linnaeus, 1758 | LC | - | - | - | - | - |
| Lepidoptera | Butterfly | Pieridae | *Anthocharis cardamines* Linnaeus, 1758 | LC | - | - | - | - | - |
| Lepidoptera | Butterfly | Pieridae | *Aporia crataegi* Linnaeus, 1758 | LC | - | - | - | - | - |
| Lepidoptera | Butterfly | Pieridae | *Colias croceus* Geoffroy, 1785 | LC | - | - | - | - | - |
| Lepidoptera | Butterfly | Pieridae | *Euchloe ausonia* Hubner, 1805 | LC | - | - | - | - | - |
| Lepidoptera | Butterfly | Pieridae | *Euchloe insularis* Staudinger, 1861 | LC | - | - | - | - | - |
| Lepidoptera | Butterfly | Pieridae | *Gonepteryx cleopatra* Linnaeus, 1767 | LC | - | - | - | - | - |
| Lepidoptera | Butterfly | Pieridae | *Pieris balcana* Lorkovic, 1970 | LC | - | - | - | - | - |
| Lepidoptera | Butterfly | Pieridae | *Pieris ergane* Geyer, 1828 | LC | - | - | - | - | - |
| Lepidoptera | Butterfly | Pieridae | *Pieris napi* Linnaeus, 1758 | LC | - | - | - | - | - |
| Lepidoptera | Butterfly | Pieridae | *Pieris rapae* Linnaeus, 1758 | LC | - | - | - | - | - |
| Lepidoptera | Moth | Erebidae | *Amata phegea* Linnaeus, 1758 | - | - | - | - | - | - |
| Lepidoptera | Moth | Erebidae | *Euclidia glyphica* Linnaeus, 1758 | - | - | - | - | - | - |
| Lepidoptera | Moth | Erebidae | *Euplagia quadripunctaria* Poda, 1761 | - | - | - | - | - | - |
| Lepidoptera | Moth | Glyphipterigidae | *Glyphipterix simpliciella* Stephens, 1834 | - | - | - | - | - | - |
| Lepidoptera | Moth | Noctuidae | *Tyta luctuosa* Denis & Schiffermüller, 1775 | - | - | - | - | - | - |
| Lepidoptera | Moth | Scythrididae | *Scythris knochella* Fabricius, 1794 | - | - | - | - | - | - |
| Lepidoptera | Moth | Sphingidae | *Hemaris fuciformis* Linnaeus, 1758 | - | - | - | - | - | - |
| Lepidoptera | Moth | Sphingidae | *Macroglossum stellatarum* Linnaeus, 1758 | - | - | - | - | - | - |
| Lepidoptera | Moth | Thyrididae | *Thyris fenestrella* Scopoli, 1763 | - | - | - | - | - | - |
| Lepidoptera | Moth | Zygaenidae | *Zygaena trifolii* Esper, 1783 | - | - | - | - | - | - |

**Table S4.** Percentage of users’ correct identification for pollinators at the functional group, genus, and species.

|  | **Group Level**  **(*n* = 872)** | | **Genus Level**  **(*n* = 584)** | | **Species Level**  **(*n* = 317)** | |
| --- | --- | --- | --- | --- | --- | --- |
|  |  |  |  |  |  |  |
|  | ***n*** | **% Corr.** | ***N*** | **% Corr.** | ***n*** | **% Corr.** |
| Butterflies | 102 | 94.1% | 75 | 92.0% | 65 | 86.2% |
| Other Groups | 770 | 93.6% | 509 | 87.8% | 252 | 86.9% |
| Difference in %s |  | +0.5 |  | +4.2 |  | −0.8 |
| *P*-value |  | 1.000 |  | 0.532 |  | 0.938 |
| Moths | 15 | 86.7% | 12 | 100.0% | 10 | 100.0% |
| Other Groups | 857 | 93.8% | 572 | 88.1% | 307 | 86.3% |
| Difference in %s |  | −7.1 |  | +11.9 |  | +13.7 |
| *P*-value |  | 0.802 |  | 0.532 |  | 0.618 |
| Bee flies | 15 | 93.3% | 12 | 91.7% | 1 | 100.0% |
| Other Groups | 857 | 93.7% | 572 | 88.3% | 316 | 86.7% |
| Difference in %s |  | −0.4 |  | +3.4 |  | +13.3 |
| *P*-value |  | 1.000 |  | 1.000 |  | 1.000 |
| Hoverflies | 109 | 92.7% | 73 | 93.2% | 49 | 91.8% |
| Other Groups | 763 | 93.8% | 511 | 87.7% | 268 | 85.8% |
| Difference in %s |  | −1.2 |  | +5.5 |  | +6.0 |
| *P*-value |  | 1.000 |  | 0.532 |  | 0.618 |
| Bees | 367 | 94.0% | 251 | 88.0% | 92 | 93.5% |
| Other Groups | 505 | 93.5% | 333 | 88.6% | 225 | 84.0% |
| Difference in %s |  | +0.5 |  | −0.5 |  | +9.5 |
| *P*-value |  | 1.000 |  | 0.917 |  | 0.091 |
| Wasps | 23 | 87.0% | 16 | 81.3% | 7 | 100.0% |
| Other Groups | 849 | 93.9% | 568 | 88.6% | 310 | 86.5% |
| Difference in %s |  | −6.9 |  | −7.3 |  | +13.5 |
| *P*-value |  | 0.802 |  | 0.532 |  | 0.693 |
| Beetles | 241 | 94.6% | 145 | 84.1% | 93 | 75.3% |
| Other Groups | 631 | 93.3% | 439 | 89.7% | 224 | 91.5% |
| Difference in %s |  | +1.3 |  | −5.6 |  | −16.2 |
| *P*-value |  | 1.000 |  | 0.476 |  | **0.001*** |

*Notes:* Observations marked as “don’t know” or left blank by users were excluded from the analysis.

**P*-value ≤0.05.

**Table S5**. List of identified plant species. The table presents the following information: Family, Species binomial, IUCN Red List classification at the European level, ASE=Alien Status Assessment, AR=Alien Range, ASAS=Alien Status Assessment Source, PPT=Primary Pathway Type as listed in the EASIN database, ISA=Invasive Status Assessment in Europe, and ISES=source of the Invasive Status Evaluation. Identifications at the genus level are provided only when a single occurrence is recorded.

| FAMILY | SPECIES | IUCN EU | A S E | A R | A S A S | P P T | I S A | I S E S |
| --- | --- | --- | --- | --- | --- | --- | --- | --- |
| Aizoaceae | *Mesembryanthemum cordifolium* L.f. | - | YES | EU | a, c | 9 | - | - |
| Aizoaceae | *Oscularia deltoides* (L.) Schwantes | - | - | - | - | - | - | - |
| Aizoaceae | *Carpobrotus edulis* (L.) N.E.Br. | - | YES | EU | b, a, c | 9, 3 | - | - |
| Amaranthaceae | *Amaranthus cruentus* L. | - | (no location) | - | - | - | - | - |
| Amaryllidaceae | *Allium ducissae* Bartolucci, Iocchi & F.Conti | NT | - | - | - | - | - | - |
| Amaryllidaceae | *Allium nigrum* L. | LC | - | - | - | - | - | - |
| Amaryllidaceae | *Narcissus tazetta* L. | - | - | - | - | - | - | - |
| Amaryllidaceae | *Notoscordium sp.* | - | - | - | - | - | - | - |
| Apiaceae | *Crithmum maritimum* L. | LC | - | - | - | - | - | - |
| Apiaceae | *Daucus carota* L. | LC | - | - | - | - | - | - |
| Apiaceae | *Ferula communis* L. | - | - | - | - | - | - | - |
| Apiaceae | *Foeniculum vulgare* Mill. | LC | - | - | - | - | - | - |
| Apiaceae | *Oenanthe crocata* L. | LC | - | - | - | - | - | - |
| Apiaceae | *Oenanthe pimpinelloides* L. | - | - | - | - | - | - | - |
| Apiaceae | *Smyrnium* sp. | - | - | - | - | - | - | - |
| Apiaceae | *Thapsia villosa* L. | - | - | - | - | - | - | - |
| Apiaceae | *Tordylium apulum*L. | - | - | - | - | - | - | - |
| Apocynaceae | *Mandevilla* sp. | - | - | - | - | - | - | - |
| Apocynaceae | *Nerium oleander* L. | LC | - | - | - | - | - | - |
| Apocynaceae | *Vinca major* L. | - | - | - | - | - | - | - |
| Araliaceae | *Hedera helix* L. | LC | - | - | - | - | - | - |
| Asparagaceae | *Ornithogalum* sp. | - | - | - | - | - | - | - |
| Asparagaceae | *Scilla* sp. | - | - | - | - | - | - | - |
| Asphodelaceae | *Asphodelus fistulosus* L. | - | - | - | - | - | - | - |
| Asphodelaceae | *Asphodelus lusitanicus* Cout. | - | - | - | - | - | - | - |
| Asphodelaceae | *Asphodelus ramosus* L. | LC | - | - | - | - | - | - |
| Asphodelaceae | *Simethis mattiazzii* (Vand.) Sacc. | - | - | - | - | - | - | - |
| Asteraceae | *Achillea* sp. | - | - | - | - | - | - | - |
| Asteraceae | *Anacyclus* sp. | - | - | - | - | - | - | - |
| Asteraceae | *Andryala integrifolia* L. | - | - | - | - | - | - | - |
| Asteraceae | *Anthemis arvensis* L. | - | - | - | - | - | - | - |
| Asteraceae | *Anthemis chia* L. | - | - | - | - | - | - | - |
| Asteraceae | *Bellis perennis* L. | - | - | - | - | - | - | - |
| Asteraceae | *Calendula arvensis* L. | - | - | - | - | - | - | - |
| Asteraceae | *Carlina* sp. | - | - | - | - | - | - | - |
| Asteraceae | *Centaurea cyanus* L. | LC | - | - | - | - | - | - |
| Asteraceae | *Centaurea nigrescens* Willd. | - | - | - | - | - | - | - |
| Asteraceae | *Centaurea spinosa* L. | - | - | - | - | - | - | - |
| Asteraceae | *Chrysanthemum* sp. | - | - | - | - | - | - | - |
| Asteraceae | *Cichorium intybus* L. | - | - | - | - | - | - | - |
| Asteraceae | *Cirsium filipendulum* Lange | - | - | - | - | - | - | - |
| Asteraceae | *Coleostephus myconis* (L.) Rchb.f. | - | - | - | - | - | - | - |
| Asteraceae | *Cosmos* sp. | - | - | - | - | - | - | - |
| Asteraceae | *Crepis micrantha* Czerep. | - | - | - | - | - | - | - |
| Asteraceae | *Crepis rubra* L. | - | - | - | - | - | - | - |
| Asteraceae | *Cynara cardunculus* L. | LC | - | - | - | - | - | - |
| Asteraceae | *Dahlia* sp. | - | - | - | - | - | - | - |
| Asteraceae | *Eupatorium cannabinum* L. | - | - | - | - | - | - | - |
| Asteraceae | *Galactites tomentosus* Moench | - | - | - | - | - | - | - |
| Asteraceae | *Gerbera* sp. | - | - | - | - | - | - | - |
| Asteraceae | *Glebionis coronaria* (L.) Cass. ex Spach | - | - | - | - | - | - | - |
| Asteraceae | *Helichrysum foetidum* (L.) Moench | - | - | - | - | - | - | - |
| Asteraceae | *Inula* sp. | - | - | - | - | - | - | - |
| Asteraceae | *Leontodon* sp. | - | - | - | - | - | - | - |
| Asteraceae | *Leucanthemum sylvaticum subsp. merinoi* (Vogt & Castrov.) Vogt & Oberpr. | - | - | - | - | - | - | - |
| Asteraceae | *Matricaria chamomilla* L. | LC | - | - | - | - | - | - |
| Asteraceae | *Montanoa bipinnatifida* (Kunth) K.Koch | - | - | - | - | - | - | - |
| Asteraceae | *Onopordum* sp. | - | - | - | - | - | - | - |
| Asteraceae | *Osteospermum* sp. | - | - | - | - | - | - | - |
| Asteraceae | *Pallenis spinosa* (L.) Cass. | - | - | - | - | - | - | - |
| Asteraceae | *Petasites pyrenaicus* (Loefl.) G.López | - | - | - | - | - | - | - |
| Asteraceae | *Picris* sp. | - | - | - | - | - | - | - |
| Asteraceae | *Pulicaria odora* (L.) Rchb. | - | - | - | - | - | - | - |
| Asteraceae | *Reichardia picroides* (L.) Roth | - | - | - | - | - | - | - |
| Asteraceae | *Scolymus hispanicus* L. | - | - | - | - | - | - | - |
| Asteraceae | *Scorzonera laciniata* L. | - | - | - | - | - | - | - |
| Asteraceae | *Jacobaea vulgaris* Gaertn. | - | - | - | - | - | - | - |
| Asteraceae | *Silybum marianum* (L.) Gaertn. | LC | - | - | - | - | - | - |
| Asteraceae | *Sonchus* sp. | - | - | - | - | - | - | - |
| Asteraceae | *Tagetes* sp. | - | - | - | - | - | - | - |
| Asteraceae | *Tanacetum* sp. | - | - | - | - | - | - | - |
| Asteraceae | *Taraxacum officinale* F.H.Wigg. | LC | - | - | - | - | - | - |
| Asteraceae | *Tragopogon* sp. | - | - | - | - | - | - | - |
| Asteraceae | *Arctotheca calendula* (L.) K.Lewin | - | YES | EU | a, c | 9, 6 | - | - |
| Asteraceae | *Calendula officinalis* L. | - | YES | EU¹ | a, c | 9, 4, 5 | - | - |
| Asteraceae | *Erigeron* sp. | - | - | - | - | - | - | - |
| Asteraceae | *Solidago gigantea* Aiton | - | YES | EU | a, c | 9 | - | - |
| Asteraceae | *Zinnia elegans* Jacq. | - | YES | EU | a, c | 9, 4 | - | - |
| Asteraceae | *Dimorphotheca pluvialis* (L.) Moench | - | YES | EU | c | NA | - | - |
| Asteraceae | *Helianthus annuus* L. | - | YES | EU | a, c | 9, 4, 5, 7 | - | - |
| Bignoniaceae | *Podranea ricasoliana* (Tanfani) Sprague | - | YES | EU | a, c | 9, 4 | - | - |
| Boraginaceae | *Anchusa* sp. | - | - | - | - | - | - | - |
| Boraginaceae | *Borago officinalis* L. | LC | - | - | - | - | - | - |
| Boraginaceae | *Echium italicum* L. | - | - | - | - | - | - | - |
| Boraginaceae | *Echium vulgare* L. | - | - | - | - | - | - | - |
| Boraginaceae | *Glandora prostrata* (Loisel.) D.C.Thomas | - | - | - | - | - | - | - |
| Boraginaceae | *Heliotropium europaeum* L. | - | - | - | - | - | - | - |
| Boraginaceae | *Symphytum bulbosum* K.F.Schimp. | - | - | - | - | - | - | - |
| Boraginaceae | *Phacelia tanacetifolia* Benth. | - | YES | EU | a, c | 1 | - | - |
| Boraginaceae | *Echium strictum subsp. strictum* L.f. | - | YES | EU² | c | NA | - | - |
| Boraginaceae | *Echium candicans* L.f. | - | YES | EU³ | c | NA | - | - |
| Brassicaceae | *Alliaria petiolata* (M.Bieb.) Cavara & Grande | - | - | - | - | - | - | - |
| Brassicaceae | *Cakile maritima* Scop. | - | - | - | - | - | - | - |
| Brassicaceae | *Capsella bursa-pastoris* (L.) Medik. | - | - | - | - | - | - | - |
| Brassicaceae | *Diplotaxis muralis* (L.) DC. | LC | - | - | - | - | - | - |
| Brassicaceae | *Diplotaxis tenuifolia* (L.) DC. | LC | - | - | - | - | - | - |
| Brassicaceae | *Erucastrum* sp. | - | - | - | - | - | - | - |
| Brassicaceae | *Erysimum* sp. | - | - | - | - | - | - | - |
| Brassicaceae | *Hirschfeldia incana* (L.) Lagr.-Foss. | - | - | - | - | - | - | - |
| Brassicaceae | *Iberis sempervirens* L. | - | - | - | - | - | - | - |
| Brassicaceae | *Isatis tinctoria* L. | LC | - | - | - | - | - | - |
| Brassicaceae | *Lepidium* sp. | - | - | - | - | - | - | - |
| Brassicaceae | *Lobularia maritima* (L.) Desv. | - | - | - | - | - | - | - |
| Brassicaceae | *Malcolmia flexuosa* (Sm.) Sm. | - | - | - | - | - | - | - |
| Brassicaceae | *Raphanus raphanistrum subsp. Raphanistrum* L. | LC | - | - | - | - | - | - |
| Brassicaceae | *Rorippa sylvestris* (L.) Besser | LC | - | - | - | - | - | - |
| Brassicaceae | *Pseudoturritis turrita* (L.) Al-Shehbaz | - | - | - | - | - | - | - |
| Butomaceae | *Butomus umbellatus* L. | LC | - | - | - | - | - | - |
| Cactaceae | *Opuntia ficus-indica* (L.) Mill. | - | YES | EU | a, c | 9, 4, 3 | - | - |
| Campanulaceae | *Campanula dichotoma* L. | - | - | - | - | - | - | - |
| Campanulaceae | *Campanula lusitanica* Loefl. | - | - | - | - | - | - | - |
| Campanulaceae | *Jasione montana* L. | - | - | - | - | - | - | - |
| Capparaceae | *Capparis spinosa* L. | - | - | - | - | - | - | - |
| Caprifoliaceae | *Cephalaria transsylvanica* (L.) Roem. & Schult. | - | - | - | - | - | - | - |
| Caprifoliaceae | *Knautia arvensis* (L.) Coult. | - | - | - | - | - | - | - |
| Caprifoliaceae | *Knautia integrifolia* (Honck. ex L.) Bertol. | - | - | - | - | - | - | - |
| Caprifoliaceae | *Lomelosia* sp. | - | - | - | - | - | - | - |
| Caprifoliaceae | *Lonicera periclymenum* L. | - | - | - | - | - | - | - |
| Caprifoliaceae | *Pycnocomon rutifolium* (Vahl) Hoffmanns. & Link | - | - | - | - | - | - | - |
| Caprifoliaceae | *Scabiosa* sp. | - | - | - | - | - | - | - |
| Caprifoliaceae | *Valeriana calcitrapae* L. | - | - | - | - | - | - | - |
| Caprifoliaceae | *Valeriana rubra* L. | - | - | - | - | - | - | - |
| Caryophyllaceae | *Silene latifolia* Poir. | - | - | - | - | - | - | - |
| Caryophyllaceae | *Silene succulenta subsp. corsica* (DC.) Nyman | - | - | - | - | - | - | - |
| Cistaceae | *Cistus creticus* L. | - | - | - | - | - | - | - |
| Cistaceae | *Cistus inflatus* Pourr. ex J.-P.Demoly | LC | - | - | - | - | - | - |
| Cistaceae | *Cistus × laxus* Aiton | LC | - | - | - | - | - | - |
| Cistaceae | *Cistus salviifolius* L. | - | - | - | - | - | - | - |
| Cistaceae | *Tuberaria guttata* (L.) Fourr. | - | - | - | - | - | - | - |
| Commelinaceae | *Commelina*  sp. | - | - | - | - | - | - | - |
| Convolvulaceae | *Calystegia* sp. | - | - | - | - | - | - | - |
| Convolvulaceae | *Convolvulus althaeoides* L. | - | - | - | - | - | - | - |
| Convolvulaceae | *Convolvulus arvensis* L. | - | - | - | - | - | - | - |
| Convolvulaceae | *Convolvulus tricolor* L. | - | - | - | - | - | - | - |
| Cornaceae | *Cornus mas* L. | LC | - | - | - | - | - | - |
| Cornaceae | *Cornus sanguinea* L. | - | - | - | - | - | - | - |
| Crassulaceae | *Sedum arenarium* Brot. | - | - | - | - | - | - | - |
| Crassulaceae | *Aeonium arboreum* (L.) Webb & Berthel. | - | YES | EU | a, c | 9 | - | - |
| Cucurbitaceae | *Bryonia* sp. | - | - | - | - | - | - | - |
| Cucurbitaceae | *Cucumis sativus* L. | - | - | - | - | - | - | - |
| Cucurbitaceae | *Ecballium elaterium* (L.) A.Rich. | - | - | - | - | - | - | - |
| Droseraceae | *Drosera binata* Labill. | - | - | - | - | - | - | - |
| Ericaceae | *Arbutus unedo* L. | LC | - | - | - | - | - | - |
| Ericaceae | *Erica carnea* L. | - | - | - | - | - | - | - |
| Ericaceae | *Erica multiflora* L. | - | - | - | - | - | - | - |
| Euphorbiaceae | *Euphorbia characias* L. | - | - | - | - | - | - | - |
| Euphorbiaceae | *Mercurialis* sp. | - | - | - | - | - | - | - |
| Euphorbiaceae | *Euphorbia pulcherrima* Willd. ex Klotzsch | - | YES | EU | a, c | NA | - | - |
| Euphorbiaceae | *Ricinus communis* L. | - | YES | EU | a, c | 9, 1 | - | - |
| Fabaceae | *Anthyllis hermanniae* L. | - | - | - | - | - | - | - |
| Fabaceae | *Anthyllis vulneraria* L. | - | - | - | - | - | - | - |
| Fabaceae | *Bituminaria bituminosa* (L.) C.H.Stirt. | - | - | - | - | - | - | - |
| Fabaceae | *Cytisus scoparius subsp. insularis* (S.Ortiz & Pulgar) Auvray | - | - | - | - | - | - | - |
| Fabaceae | *Erophaca* sp. | - | - | - | - | - | - | - |
| Fabaceae | *Hippocrepis emerus subsp. emerus* (L.) Lassen | - | - | - | - | - | - | - |
| Fabaceae | *Lathyrus* sp. | - | - | - | - | - | - | - |
| Fabaceae | *Lotus dorycnium* L. | LC | - | - | - | - | - | - |
| Fabaceae | *Lotus tetragonolobus* L. | - | - | - | - | - | - | - |
| Fabaceae | *Lupinus angustifolius* L. | - | - | - | - | - | - | - |
| Fabaceae | *Medicago arborea* L. | - | - | - | - | - | - | - |
| Fabaceae | *Melilotus* sp. | - | - | - | - | - | - | - |
| Fabaceae | *Onobrychis* sp. | - | - | - | - | - | - | - |
| Fabaceae | *Spartium junceum* L. | - | - | - | - | - | - | - |
| Fabaceae | *Sulla coronaria* (L.) B.H.Choi & H.Ohashi | LC | - | - | - | - | - | - |
| Fabaceae | *Trifolium cherleri* L. | - | - | - | - | - | - | - |
| Fabaceae | *Trifolium medium* L. | - | - | - | - | - | - | - |
| Fabaceae | *Trifolium nigrescens* Viv. | LC | - | - | - | - | - | - |
| Fabaceae | *Trifolium repens* L. | LC | - | - | - | - | - | - |
| Fabaceae | *Trigonella balansae* Boiss. & Reut. | - | - | - | - | - | - | - |
| Fabaceae | *Ulex europaeus* L. | LC | - | - | - | - | - | - |
| Fabaceae | *Vicia faba* L. | - | - | - | - | - | - | - |
| Fabaceae | *Vicia hybrida* L. | LC | - | - | - | - | - | - |
| Fabaceae | *Vicia villosa* Roth | - | - | - | - | - | - | - |
| Fabaceae | *Wisteria sp.* | - | YES | EU | c | - | - | - |
| Fabaceae | *Amorpha fruticosa* L. | - | YES | EU | a, c | 9 | - | - |
| Geraniaceae | *Erodium malacoides* (L.) L'Hér. | - | - | - | - | - | - | - |
| Geraniaceae | *Erodium moschatum* (L.) L'Hér. | - | - | - | - | - | - | - |
| Geraniaceae | *Geranium columbinum* L. | - | - | - | - | - | - | - |
| Geraniaceae | *Geranium molle* L. | - | - | - | - | - | - | - |
| Geraniaceae | *Pelargonium* sp. | - | - | - | - | - | - | - |
| Hypericaceae | *Hypericum tetrapterum* Fr. | - | - | - | - | - | - | - |
| Lamiaceae | *Ajuga reptans* L. | - | - | - | - | - | - | - |
| Lamiaceae | *Clinopodium nepeta* (L.) Kuntze | - | - | - | - | - | - | - |
| Lamiaceae | *Lamium amplexicaule* L. | - | - | - | - | - | - | - |
| Lamiaceae | *Lamium garganicum* L. | - | - | - | - | - | - | - |
| Lamiaceae | *Lamium maculatum* (L.) L. | - | - | - | - | - | - | - |
| Lamiaceae | *Lamium purpureum* L. | - | - | - | - | - | - | - |
| Lamiaceae | *Mentha aquatica* L. | - | - | - | - | - | - | - |
| Lamiaceae | *Nepeta cataria* L. | LC | - | - | - | - | - | - |
| Lamiaceae | *Origanum majorana* L. | - | - | - | - | - | - | - |
| Lamiaceae | *Origanum onites* L. | LC | - | - | - | - | - | - |
| Lamiaceae | *Origanum vulgare* L. | LC | - | - | - | - | - | - |
| Lamiaceae | *Phlomis fruticosa* L. | - | - | - | - | - | - | - |
| Lamiaceae | *Prasium majus* L. | - | - | - | - | - | - | - |
| Lamiaceae | *Pseudodictamnus acetabulosus* (L.) Salmaki & Siadati | - | - | - | - | - | - | - |
| Lamiaceae | *Salvia glutinosa* L. | - | - | - | - | - | - | - |
| Lamiaceae | *Salvia leucantha* Cav. | - | - | - | - | - | - | - |
| Lamiaceae | *Salvia officinalis* L. | LC | - | - | - | - | - | - |
| Lamiaceae | *Salvia rosmarinus* Spenn. | LC | - | - | - | - | - | - |
| Lamiaceae | *Teucrium fruticans* L. | - | - | - | - | - | - | - |
| Lamiaceae | *Vitex agnus-castus* L. | DD | NO | - | - | - | - | - |
| Lamiaceae | *Lavandula angustifolia* Mill. | LC | - | - | - | - | - | - |
| Lamiaceae | *Ocimum basilicum* L. | - | YES | EU | a, c | 9, 4, 1 | - | - |
| Lythraceae | *Punica granatum* L. | - | YES | EU | a, c | 9, 4, 1 | - | - |
| Malvaceae | *Abutilon grandiflorum* G.Don | - | - | - | - | - | - | - |
| Malvaceae | *Dombeya wallichii* (Lindl.) Benth. ex Baill. | - | - | - | - | - | - | - |
| Malvaceae | *Hibiscus*sp. | - | - | - | - | - | - | - |
| Malvaceae | *Kosteletzkya pentacarpos* (L.) Ledeb. | VU | - | - | - | - | - | - |
| Malvaceae | *Malva arborea* (L.) Webb & Berthel. | - | - | - | - | - | - | - |
| Malvaceae | *Malva moschata* L. | - | - | - | - | - | - | - |
| Malvaceae | *Malva multiflora* (Cav.) Soldano, Banfi & Galasso | - | - | - | - | - | - | - |
| Malvaceae | *Malva sylvestris* L. | LC | - | - | - | - | - | - |
| Malvaceae | *Tilia* sp. | - | - | - | - | - | - | - |
| Malvaceae | *Ceiba speciosa* (A.St.-Hil., A.Juss. & Cambess.) Ravenna | - | YES | EU | a, c | 9,1 | - | - |
| Myrtaceae | *Metrosideros excelsa* Sol. ex Gaertn. | - | - | - | - | - | - | - |
| Myrtaceae | *Myrtus communis* L. | LC | - | - | - | - | - | - |
| Nyctaginaceae | *Bougainvillea glabra* Choisy | LC | YES | EU | a, c | 9, 4 | - | - |
| Oleaceae | *Jasminum* sp. | - | - | - | - | - | - | - |
| Oleaceae | *Olea europaea* L. | DD | - | - | - | - | - | - |
| Oleaceae | *Ligustrum sinense* Lour. | - | YES | EU | a, c | 9 | - | - |
| Orchidaceae | *Anacamptis* sp. | - | - | - | - | - | - | - |
| Orchidaceae | *Gymnadenia widderi* (Teppner & E.Klein) Teppner & E.Klein | EN | - | - | - | - | - | - |
| Orchidaceae | *Ophrys sphegodes subsp. helenae* (Renz) Soó & D.M.Moore | LC | - | - | - | - | - | - |
| Orobanchaceae | *Parentucellia viscosa (L.)* Caruel | - | - | - | - | - | - | - |
| Oxalidaceae | *Oxalis acetosella* L. | - | - | - | - | - | - | - |
| Oxalidaceae | *Oxalis debilis* Kunth | - | YES | EU | a, c | 7, 8 | - | - |
| Oxalidaceae | *Oxalis pes-caprae* L. | - | YES | EU | b, a, c | 9, 7 | Yes | ISSG |
| Papaveraceae | *Chelidonium majus* L. | LC | - | - | - | - | - | - |
| Papaveraceae | *Fumaria capreolata* L. | - | - | - | - | - | - | - |
| Papaveraceae | *Fumaria officinalis* L. | - | - | - | - | - | - | - |
| Papaveraceae | *Glaucium flavum* Crantz | LC | - | - | - | - | - | - |
| Papaveraceae | *Papaver rhoeas* L. | LC | - | - | - | - | - | - |
| Papaveraceae | *Papaver somniferum* L. | LC | - | - | - | - | - | - |
| Passifloraceae | *Passiflora caerulea* L. | - | YES | EU | a, c | 4, 9 | - | - |
| Pittosporaceae | *Pittosporum* sp. | - | - | - | - | - | - | - |
| Plantaginaceae | *Antirrhinum siculum* Mill. | - | - | - | - | - | - | - |
| Plantaginaceae | *Digitalis purpurea* L. | LC | - | - | - | - | - | - |
| Plantaginaceae | *Linaria reflexa* (L.) Chaz. | - | - | - | - | - | - | - |
| Plantaginaceae | *Misopates orontium* (L.) Raf. | - | - | - | - | - | - | - |
| Plantaginaceae | *Plantago* sp. | - | - | - | - | - | - | - |
| Plantaginaceae | *Veronica persica* Poir. | - | - | - | - | - | - | - |
| Plantaginaceae | *Antirrhinum majus* L. | - | YES | EU⁴ | c | NA | - | - |
| Plantaginaceae | *Cymbalaria muralis* G.Gaertn., B.Mey. & Scherb. | - | - | - | - | - | - | - |
| Plumbaginaceae | *Armeria* sp. | - | - | - | - | - | - | - |
| Plumbaginaceae | *Limonium* sp. | - | - | - | - | - | - | - |
| Polygonaceae | *Polygonum scoparium* Req. ex Loisel. | - | - | - | - | - | - | - |
| Portulacaceae | *Portulaca oleracea* L. | - | - | - | - | - | - | - |
| Primulaceae | *Cyclamen* sp. | - | - | - | - | - | - | - |
| Primulaceae | *Lysimachia arvensis* (L.) U.Manns & Anderb. | - | - | - | - | - | - | - |
| Ranunculaceae | *Aconitum napellus* L. | LC | - | - | - | - | - | - |
| Ranunculaceae | *Anemone* sp. | - | - | - | - | - | - | - |
| Ranunculaceae | *Helleborus* sp. | - | - | - | - | - | - | - |
| Ranunculaceae | *Nigella* sp. | - | - | - | - | - | - | - |
| Ranunculaceae | *Ranunculus acris* L. | - | - | - | - | - | - | - |
| Ranunculaceae | *Ranunculus bulbosus* L. | - | - | - | - | - | - | - |
| Ranunculaceae | *Ranunculus ficaria* L. | LC | - | - | - | - | - | - |
| Ranunculaceae | *Ranunculus macrophyllus* Dsf. | LC | - | - | - | - | - | - |
| Resedaceae | *Reseda alba* L. | - | - | - | - | - | - | - |
| Rhamnaceae | *Rhamnus alaternus* L. | LC | - | - | - | - | - | - |
| Rosaceae | *Cotoneaster* sp. | - | - | - | - | - | - | - |
| Rosaceae | *Malus* sp. | - | - | - | - | - | - | - |
| Rosaceae | *Potentilla* sp. | - | - | - | - | - | - | - |
| Rosaceae | *Prunus* sp. | - | - | - | - | - | - | - |
| Rosaceae | *Pyrus* sp. | - | - | - | - | - | - | - |
| Rosaceae | *Rhaphiolepis indica* (L.) Lindl. | LC | - | - | - | - | - | - |
| Rosaceae | *Rosa* sp. | - | - | - | - | - | - | - |
| Rosaceae | *Rubus ulmifolius* J.Presl & C.Presl | - | - | - | - | - | - | - |
| Rosaceae | *Crataegus germanica* (L.) Kuntze | - | YES | EU | a, c | 9, 2 | - | - |
| Rosaceae | *Chaenomeles japonica* (Thunb.) Lindl. ex Spach | - | YES | EU | a, c | 9, 4 | - | - |
| Rutaceae | *Citrus × aurantium f. aurantium* L. | - | YES | EU | a, c | NA | - | - |
| Rutaceae | *Citrus reticulata* Blanco | - | YES | EU | a, c | NA | - | - |
| Salicaceae | *Salix* sp. | - | - | - | - | - | - | - |
| Solanaceae | *Cestrum* sp. | - | - | - | - | - | - | - |
| Solanaceae | *Solanum nigrum* L. | - | - | - | - | - | - | - |
| Strelitziaceae | *Strelitzia* sp. | - | - | - | - | - | - | - |
| Tamaricaceae | *Tamarix chinensis* Lour. | - | YES | EU | a, c | 9 | - | - |
| Thymelaeaceae | *Daphne gnidium* L. | - | - | - | - | - | - | - |
| Tropaeolaceae | *Tropaeolum majus* L. | - | YES | EU | a, c | 9, 4, 5 | - | - |
| Verbenaceae | *Phyla nodiflora* (L.) Greene | LC | - | - | - | - | - | - |
| Verbenaceae | *Lantana camara* L. | - | YES | EU | a, c | 9 | - | - |
| Viburnaceae | *Viburnum* sp. | - | - | - | - | - | - | - |
| Violaceae | *Viola odorata* L. | LC | - | - | - | - | - | - |

A R = ¹ except Spain, ² except Canary islands, ³ except Madeira, ⁴ except Spain and France.

A S E S = a: European Alien Species Information Network (<https://easin.jrc.ec.europa.eu/spexplorer>); b: Global Invasive Species Database (<https://www.iucngisd.org/gisd>); c: Plants of the World Online ([https://powo.science.kew.org](https://powo.science.kew.org/)).

P P T = 1: Agriculture; 2: Botanic garden; 3: Erosion control; 4: Horticulture; 5: Improvement in the wild landscape; 6: Seed contaminant; 7: Transport; 8: Unaided; 9: Ornamental.

**Table S6.** Percentage of users’ correct identification of lants at the family, genus, and species level, overall and by family.

|  | **Family Level** | | | **Genus Level** | | | **Species Level** | | |
| --- | --- | --- | --- | --- | --- | --- | --- | --- | --- |
|  | (n = 741) | | | (n = 571) | | | (n = 383) | | |
|  | n | % Corr. | P-value | n | % Corr. | P-value | n | % Corr. | P-value |
| All | 741 | 96% | · | 571 | 87% | - | 383 | 78% | - |
| Aizoaceae | 4 | 75% | 1 | 3 | 100% | 1 | 2 | 100% | 1 |
| Amaranthaceae | 0 | - | - | 0 | - | - | 0 | - | - |
| Amaryllidaceae | 9 | 89% | 1 | 6 | 89% | 1 | 3 | 100% | 1 |
| Apiaceae | 28 | 96% | 1 | 15 | 100% | 1 | 12 | 100% | 1 |
| Apocynaceae | 3 | 100% | 1 | 3 | 100% | 1 | 3 | 100% | 1 |
| Araliaceae | 10 | 100% | 1 | 10 | 100% | 1 | 6 | 100% | 1 |
| Asparagaceae | 0 | - | - | 0 | - | - | 0 | - | - |
| Asphodelaceae | 6 | 83% | 1 | 5 | 83% | 1 | 1 | 20% | 0.45 |
| Asteraceae | 223 | 95% | 1 | 85 | 69% | <0.001* | 45 | 64% | 1 |
| Bignoniaceae | 0 | - | - | 0 | - | - | 0 | - | - |
| Boraginaceae | 28 | 100% | 1 | 15 | 93% | 1 | 16 | 100% | 1 |
| Brassicaceae | 29 | 97% | 1 | 12 | 75% | 1 | 9 | 77% | 1 |
| Butomaceae | 1 | 100% | 1 | 1 | 100% | 1 | 1 | 100% | 1 |
| Cactaceae | 3 | 100% | 1 | 3 | 100% | 1 | 2 | 100% | 1 |
| Campanulaceae | 3 | 100% | 1 | 3 | 100% | 1 | 2 | 100% | 1 |
| Capparaceae | 2 | 100% | 1 | 2 | 100% | 1 | 0 | - | - |
| Caprifoliaceae | 26 | 92% | 1 | 15 | 67% | 0.91 | 12 | 100% | 1 |
| Caryophyllaceae | 1 | 100% | 1 | 1 | 100% | 1 | 1 | 100% | 1 |
| Cistaceae | 46 | 98% | 1 | 44 | 98% | 1 | 7 | 71% | 1 |
| Commelinaceae | 2 | 100% | 1 | 2 | 100% | 1 | 0 | - | - |
| Convolvulaceae | 12 | 100% | 1 | 10 | 83% | 1 | 2 | 100% | 1 |
| Cornaceae | 1 | 100% | 1 | 1 | 100% | 1 | 1 | 100% | 1 |
| Crassulaceae | 5 | 100% | 1 | 5 | 100% | 1 | 0 | - | - |
| Cucurbitaceae | 4 | 100% | 1 | 4 | 100% | 1 | 3 | 100% | 1 |
| Droseraceae | 1 | 100% | 1 | 1 | 100% | 1 | 1 | 100% | 1 |
| Ericaceae | 5 | 100% | 1 | 5 | 100% | 1 | 4 | 80% | 1 |
| Euphorbiaceae | 4 | 80% | 1 | 4 | 80% | 1 | 3 | 100% | 1 |
| Fabaceae | 53 | 96% | 1 | 36 | 88% | 1 | 21 | 90% | 1 |
| Geraniaceae | 8 | 100% | 1 | 7 | 100% | 1 | 2 | 40% | 1 |
| Hypericaceae | 0 | - | - | 0 | - | - | 0 | - | - |
| Lamiaceae | 50 | 92% | 1 | 32 | 94% | 1 | 17 | 77% | 1 |
| Lythraceae | 1 | 100% | 1 | 1 | 100% | 1 | 1 | 100% | 1 |
| Malvaceae | 36 | 95% | 1 | 36 | 97% | 1 | 19 | 100% | 0.93 |
| Myrtaceae | 2 | 100% | 1 | 2 | 100% | 1 | 2 | 100% | 1 |
| Nyctaginaceae | 1 | 100% | 1 | 1 | 100% | 1 | 0 | - | - |
| Oleaceae | 3 | 100% | 1 | 3 | 100% | 1 | 1 | 100% | 1 |
| Orchidaceae | 2 | 100% | 1 | 2 | 100% | 1 | 2 | 100% | 1 |
| Orobanchaceae | 1 | 100% | 1 | 1 | 100% | 1 | 1 | 100% | 1 |
| Oxalidaceae | 19 | 100% | 1 | 19 | 100% | 1 | 3 | 18% | <0.05* |
| Papaveraceae | 19 | 95% | 1 | 18 | 95% | 1 | 3 | 100% | 1 |
| Passifloraceae | 1 | 100% | 1 | 1 | 100% | 1 | 1 | 100% | 1 |
| Pittosporaceae | 1 | 100% | 1 | 1 | 100% | 1 | 0 | - | - |
| Plantaginaceae | 9 | 100% | 1 | 9 | 100% | 1 | 6 | 100% | 1 |
| Plumbaginaceae | 1 | 100% | 1 | 1 | 100% | 1 | 0 | - | - |
| Polygonaceae | 1 | 100% | 1 | 1 | 100% | 1 | 1 | 100% | 1 |
| Portulacaceae | 1 | 100% | 1 | 1 | 100% | 1 | 0 | - | - |
| Primulaceae | 1 | 100% | 1 | 1 | 100% | 1 | 0 | - | - |
| Ranunculaceae | 15 | 100% | 1 | 15 | 100% | 1 | 8 | 100% | 1 |
| Resedaceae | 10 | 80% | 1 | 8 | 80% | 1 | 5 | 100% | 1 |
| Rhamnaceae | 1 | 100% | 1 | 1 | 100% | 1 | 1 | 100% | 1 |
| Rosaceae | 19 | 100% | 1 | 17 | 100% | 1 | 2 | 100% | 1 |
| Rutaceae | 6 | 100% | 1 | 6 | 100% | 1 | 0 | - | - |
| Salicaceae | 2 | 100% | 1 | 2 | 100% | 1 | 0 | - | - |
| Solanaceae | 3 | 100% | 1 | 3 | 100% | 1 | 2 | 100% | 1 |
| Strelitziaceae | 1 | 100% | 1 | 1 | 100% | 1 | 0 | - | - |
| Tamaricaceae | 2 | 100% | 1 | 2 | 50% | 1 | 0 | - | - |
| Thymelaeaceae | 1 | 100% | 1 | 1 | 100% | 1 | 0 | - | - |
| Tropaeolaceae | 2 | 100% | 1 | 2 | 100% | 1 | 2 | 100% | 1 |
| Verbenaceae | 7 | 100% | 1 | 7 | 100% | - | 2 | 100% | 1 |
| Viburnaceae | 0 | - | - | 0 | - | 1 | 0 | - | - |
| Violaceae | 2 | 100% | 1 | 2 | 100% | 1 | 0 | - | - |

*Notes:* Observations left blank by users were excluded from the analysis.

**P*-value <0.05.

**Table** S7. LIFE 4 Pollinators bioblitz sites. The table presents the country, region, Natura 2000 site code (if applicable), and location name.

| **Country** | **Region** | **N2000 site** | **Location** |
| --- | --- | --- | --- |
| Greece | Sigri, Lesvos | GR4110003 | Lesvos: Dytiki Chersonisos - Apolithomeno Dasos |
| Greece | Lesvos | NA | Castle of Mytilene |
| Spain | Mallorca | ES0000544 | Son Real |
| Spain | Mallorca | ES0000037 | Es Trenc - Salobrar de Campos. |
| Spain | Mallorca | ES0000226 | L'Albufereta |
| Spain | Mallorca | ES0000038 | S'Albufera de Mallorca |
| Spain | Mallorca | ES0000073 | Costa Brava de Mallorca |
| Spain | Mallorca | ES0000227 | Muntanyes d'Artà |
| Spain | Mallorca | ES0000145 | Mondragó |
| Spain | Formentera | ES0000084 | Ses Salines d'Eivissa i Formentera |
| Spain | Cies and Ons Islands, Galicia | ES0000001/ ES0000254/ES1140004 | Illas Cíes/ Illa de Ons/ Complexo Ons - O Grove |
| Spain | Galicia | ES1120001 | Ancares - Courel |
| Italy | Lombardia | IT20A0004 | Le Bine |
| Italy | Emilia-Romagna | IT4050001 | Gessi Bolognesi, Calanchi dell'Abbadessa |
| Italy | Abruzzo | IT7110128 | Parco Nazionale Gran Sasso - Monti della Laga |
| Italy | Emilia-Romagna | NA | Garden of the Mediateca, San Lazzaro di Savena (BO) |
| Italy | Tuscany | IT51A0008 | Monte d'Alma |
| San Marino | San Marino | NA | Parco Apistico |
| Slovenia | Slovenia | SI5000023 | Kras |
